# Borrelia peptidoglycan interacting Protein (BpiP) contributes to the fitness of *Borrelia burgdorferi* against host-derived factors and influences virulence in mouse models of Lyme disease

**DOI:** 10.1371/journal.ppat.1009535

**Published:** 2021-04-21

**Authors:** Yue Chen, Sean M. Vargas, Trever C. Smith, Sai Lakshmi Rajasekhar Karna, Taylor MacMackin Ingle, Karen L. Wozniak, Floyd L. Wormley, Janakiram Seshu

**Affiliations:** South Texas Center for Emerging Infectious Diseases (STCEID) and Department of Biology, The University of Texas at San Antonio, San Antonio, Texas, United States of America; University of Montana, UNITED STATES

## Abstract

The Peptidoglycan (PG) cell wall of the Lyme disease (LD) spirochete, *Borrelia burgdorferi (Bb)*, contributes to structural and morphological integrity of *Bb*; is a persistent antigen in LD patients; and has a unique pentapeptide with L-Ornithine as the third amino acid that cross-links its glycan polymers. A borrelial homolog (BB_0167) interacted specifically with borrelilal PG via its peptidoglycan interacting motif (MHELSEKRARAIGNYL); was localized to the protoplasmic cylinder of *Bb*; and was designated as Borrelia peptidoglycan interacting Protein (BpiP). A *bpiP* mutant displayed no defect under *in vitro* growth conditions with similar levels of several virulence-related proteins. However, the burden of *bpiP* mutant in C3H/HeN mice at day 14, 28 and 62 post-infection was significantly lower compared to control strains. No viable *bpiP* mutant was re-isolated from any tissues at day 62 post-infection although *bpiP* mutant was able to colonize immunodeficient SCID at day 28 post-infection. Acquisition or transmission of *bpiP* mutant by *Ixodes scapularis* larvae or nymphs respectively, from and to mice, was significantly lower compared to control strains. Further analysis of *bpiP* mutant revealed increased sensitivity to vancomycin, osmotic stress, lysosomal extracts, human antimicrobial peptide cathelicidin-LL37, complement-dependent killing in the presence of day 14 post-infection mouse serum and increased internalization of CFSC-labeled *bpiP* mutant by macrophages and dendritic cells compared to control strains. These studies demonstrate the importance of accessory protein/s involved in sustaining integrity of PG and cell envelope during different phases of *Bb* infection.

## Author summary

*Borrelia burgdorferi (Bb)*, the agent of Lyme disease, is capable of survival in widely divergent hosts such as the tick vector and numerous vertebrates. Peptidoglycan (PG) cell wall contributes to structural integrity of *Bb*, remodeling of its cell envelope in response to environmental signals and in modulation of antimicrobial responses in different hosts during the infectious cycle. The glycan polymers of PG are cross-linked via a unique pentapeptide with L-Ornithine as the third amino acid. Here, we demonstrate the role of a borrelial protein, designated as Borrelia peptidoglycan interacting Protein (BpiP), in the patho-physiology of *Bb*. Deletion of *bpiP* results in attenuation of infection in immunocompetent C3H/HeN or BALB/c mice unlike in immunodeficient SCID mice. The *bpiP* mutant is more susceptible to effects of vancomycin, osmotic stress, lysosomal extracts, antimicrobial peptides, complement-dependent killing and increased phagocytosis by macrophages and dendritic cells. These observations demonstrated that cellular and soluble factors of the host immune response limit the colonization of *bpiP* mutant in immunocompetent hosts. Understanding the role of cell wall components and its accessory factors in the survival of *Bb* during tick and mammalian phases of infection is anticipated to advance strategies to reduce the incidence of Lyme disease.

## Introduction

Lyme disease is the most common tick-borne infectious disease in the US with more than 40,000 confirmed and around 300,000 estimated infections occurring each year according to Centers for Disease Control and Prevention [1]. The causative agent of Lyme disease, *Borrelia burgdorferi (Bb)*, is a spirochetal pathogen that is transmitted to humans and other vertebrate hosts following the bite of an infected *Ixodes scapularis* tick. *Bb* has a compact genome with limited metabolic capabilities [2–4]. As an extreme auxotroph, *Bb* is constrained by 1) environmental signals; 2) limited/variable levels of key nutrients; and 3) effects of numerous anti-microbial factors impacting its survival in ticks and vertebrate hosts [2, 5]. To overcome these bottlenecks, *Bb* undergoes extensive remodeling of its cell envelope—comprising of an inner cytoplasmic membrane, the peptidoglycan (PG) cell wall and the outer membrane with a constellation of primarily lipoproteins—connecting its metabolism and survival strategies to availability/transport of nutrients to survive and colonize highly divergent hosts. The molecular mechanisms and key determinants that contribute to remodeling and structural integrity of the borrelial cell envelope facilitating host-specific adaptation of *Bb* are unclear and are open areas of investigation.

A large body of information is available on the metabolic processes and gene regulatory networks involved in the expression of the surface-exposed proteins that enable interactions of *Bb* with host cells [6–8]. Moreover, these interactions also modulate host-response to *Bb* infection and in the pathogenic mechanisms leading to colonization of *Bb* in reservoir and dead-end hosts [9]. The role of a number of transporters localized in the periplasmic space and the inner membrane connecting the external environmental milieu to metabolic processes has underscored the importance of changes in the inner membrane impacting assimilation of nutrients that are abundant or sparse during different stages of *Bb* life cycle [10–14]. Although *Bb* is a diderm, the absence of lipopolysaccharide and prevalence of a large proportion of phosphatidylcholine in place of phosphatidylethanolamine in the phospholipid bilayer of the inner and outer membranes distinguish the cell envelope of *Bb* from that of other Gram-negative organisms such as *Escherichia coli* [15, 16]. In addition, the presence of flagella in the periplasmic space confers motility and spiral structure of *Bb* [17]. It is likely that the peptidoglycan (PG) cell wall confers integrity and modulates host response during the tick and mammalian phases of infection [6].

The biogenesis and synchronization of PG synthesis with cell division and growth rates of *Bb* under different host-specific conditions are beginning to be understood in greater detail [18, 19]. The PG biosynthetic pathway starts with the influx of Short-Chain Fatty Acids (SCFAs) such as acetate that leads to the formation of key substrates such as acetyl CoA, mevalonate and lipid II critical for the biogenesis of PG [19–24]. Deletion of genes encoding acetate kinase (*ackA*) or phosphate acetyl transferase (*pta*) was lethal for *Bb*. However, mutants lacking these genes could be rescued with supplementation of mevalonate in the growth medium [23]. Recently, it has been proposed that the breakdown products of borrelial PG accumulate in the medium without being recycled and serves as a stable stimulatory component in the synovial fluid of Lyme disease patients resulting in a sustained pro-inflammatory response [25]. Therefore, a greater understanding of underlying mechanisms that contribute to the biogenesis and integrity of borrelial cell wall will advance strategies to disrupt *Bb* survival during its infectious cycle.

Previous reports have shown that spirochetes, including *B*. *burgdorferi* and Treponemes but not Leptospira spp., have unique PG with a pentapeptide comprising of L-Ornithine (along with L- and D-Alanine, D-Glutamic acid, and Glycine) that cross-links N-acetyl glucosamine (GlcNAc) and N-acetyl muramic acid (MurNAc) polymers [25–28]. The significance of L-Ornithine as a key structural difference in the PG of *Bb* in comparison to the presence of *meso*-diaminopimeleic acid (*meso*-DAP) and L-Lysine as the branched chain residues in the pentapeptide of many Gram-negative and Gram-positive bacteria, respectively, provides an avenue to dissect the molecular basis of the contribution of borrelial PG to innate and adaptive immune responses against Lyme spirochetes during different stages of infectious cycle [29].

Bioinformatic analysis of *Bb* genome revealed the presence of a homolog (BB_0167) with similarity to the C-terminal domain of OmpA-C-like proteins and with structural features suggesting its interactions with the pentapeptide of *Bb* PG [30]. OmpA-C-like homologs are prevalent in many prokaryotes and contribute to the integrity of the cell wall. The C-terminal domain of a periplasmic protein of *Neisseria meningitidis*, RmpM, shares similarities with the C-terminal domain of OmpA of *Escherichia coli* with a PG-binding function [31]. Additionally, there are examples of proteins such as Pal, a lipoprotein, and MotB, a flagellar motor protein, known to be associated with the PG. While Pal is anchored to the outer membrane of *N*. *meningitidis* via its N-terminal lipid group, the MotB protein has been shown to anchor the inner membrane to PG to immobilize the stator ring of the bacterial flagellar motor [32–34]. Moreover, *Vibrio cholerae* flagellar motor proteins, PomB and MotY, interact with inner membrane and PG [35]. Based on these aforementioned observations and in conjunction with the conserved sequence and structural features, it is feasible that BB_0167 confers stability to the cell wall in particular or enable the cell envelope to withstand a variety of stressors encountered during the infectious cycle of *Bb*. Moreover, *bb_0167* was up-regulated in fed ticks suggesting the relevance of this protein during the adaptation/survival of *Bb* during different stages of its infectious cycle [36]. The complex and unique organization of cell envelope, a distinct pentapeptide with L-Ornithine as the third amino acid cross-linking the glycan polymers, and the ability for extensive remodeling in different hosts indicate novel structure/function attributes for proteins such as BB_0167 predicted to interact with PG in the survival of *Bb* within highly disparate host.

In this study, we focused on the biochemical, genetic and phenotypic analysis of a mutant lacking BB_0167, which we designated as Borrelia peptidoglycan interacting Protein (BpiP) based on several lines of evidence and will be referred to as such for the rest of the manuscript. We localized BpiP to the protoplasmic cylinder/periplasmic fraction of *Bb* and determined its ability to bind borrelilal PG under *in vitro* conditions. Moreover, a mutant lacking *bpiP* displayed a significant colonization deficit in immunocompetent C3H/HeN and BALB/c mice at day 28 and 62 but not in the immunodeficient SCID mice. Rates of acquisition and transmission of *bpiP* mutant by *I*. *scapularis* larvae and nymphs respectively, from and to C3H/HeN mice, were significantly lower than the control strains reflecting the lower burden of *bpiP* mutant acquired from mammalian host. Phenotypic analysis of the *bpiP* mutant under *in vitro* conditions indicated increased sensitivity to select antibiotics, osmotic stress, lysosomal extracts, select antimicrobial peptides, increased phagocytosis by dendritic cells and activated macrophages and susceptibility to complement-dependent killing in the presence of serum from immunocompetent mice obtained at day 14 post-infection. Taken together, our data suggest that BpiP plays a role in facilitating *Bb* to withstand environmental stressors and soluble/cellular mediators of host immune response. These observations open avenues to exploit the novel structural and functional features of proteins that contribute to the stability of the cell wall/cell envelope to help advance strategies to limit the survival of *Bb* during the tick and mammalian phases of infection.

## Results

### Bioinformatic analysis of BpiP

**Fig 1:**
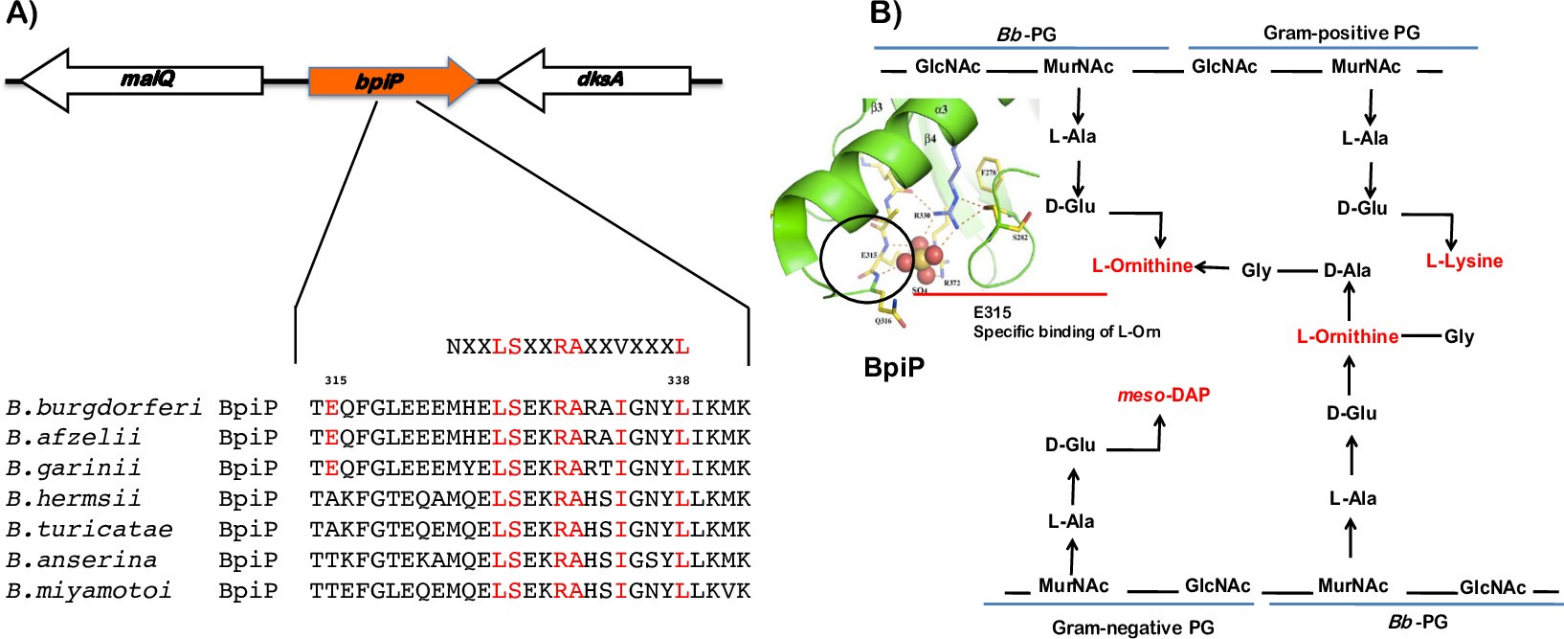
Sequence analysis of BpiP (BB_0167) and its predicted site of interaction with Peptidoglycan (PG) of *Bb*. **(A)** The location of *bpiP* (*bb_0167*) between *malQ* and *dksA* is conserved in borrelial species examined by bioinformatics analysis of the published genomes. A conserved peptidoglycan-interacting domain between amino acid positions 314 and 342 is present in all BpiP homologs in different borrelial species examined. **(B)** The PG of *Bb* is unique in having L-Ornithine as the third amino acid in the pentapeptide bridge cross-linking N-acetyl glucosamine (GlcNAc) and N-acetyl muramic acid (MurNAc) polymers compared to the presence of *meso*-DAP or L-Lysine in the PG of Gram-negative and Gram-positive bacteria, respectively. Structural analysis of BB_0167 revealed the presence of a Glutamic acid as amino acid at position 315 (E315) mediating interaction with L-Ornithine in the pentapeptide bridge. Fig 1B is adapted from references [25, 30].

Sequence analysis revealed that BpiP is a ~42 kDA (381 amino acids) protein with homology to the C-terminal region of OmpA-C-like proteins with a conserved PG interaction domain spanning the residues 315 and 338. It is predicted that this domain is involved in the formation of non-covalent interactions between BpiP and PG of *Bb* (Fig 1A). The gene encoding for BpiP is located in between *malQ* and *dksA* homologs of *Bb* within the linear chromosome and is a distinct transcriptional unit [2]. Recently, the crystal structure of BpiP was solved and compared with the C-terminal domains of OmpA-C like homologs from *Salmonella enterica* serovar *typhimurium (St)* and *Acenitobacter baumanii (Ab)* (Fig 1B, adapted and modified from [30]). Key findings relevant to the current study include the prediction that a Glutamic acid (E315) residue in BpiP potentially interacts with L-Ornithine in the *Bb* pentapeptide in place of Aspartic acid present in other OmpA-C homologs that facilitate this interaction with *meso*-DAP present in the pentapeptide of *St* or *Ab* [30]. Additionally, the crystal structure of BpiP revealed the binding of sulfate anion (SO_4_^2-^, assimilated from the crystallization buffer containing ammonium sulfate) outside of the PG binding domain interacts with a conserved residue at position R330 reflecting the bidendate salt bridges and hydrogen bond formation contributing to its interactions with PG [30]. These observations suggest the BpiP has structural features to interact with L-Ornithine that is unique to the pentapeptide crosslinks present in borrelial PG.

### Localization of BpiP in *Bb*

**Fig 2:**
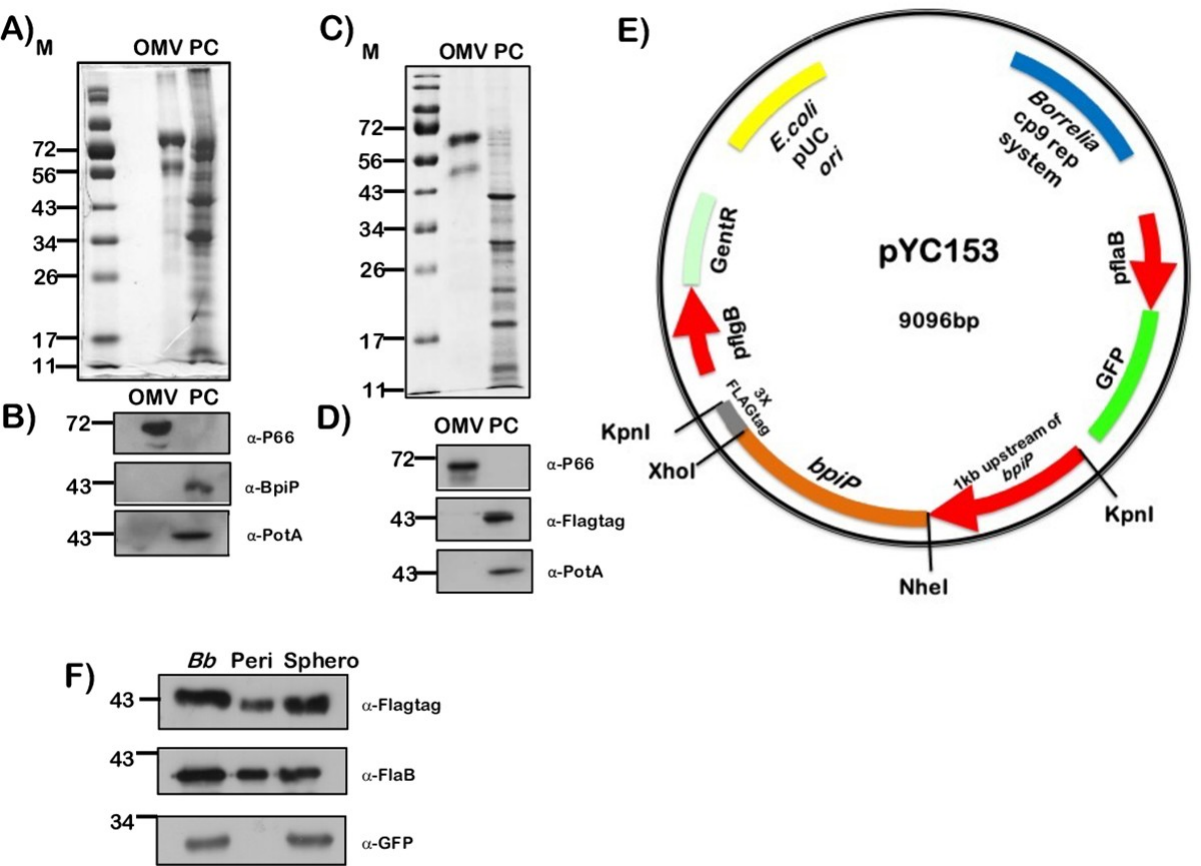
Cellular Localization of BpiP. Fractionation of (**A**) *Bb* wild type strain B31- A3 and (**C**) *bpiP* mutant expressing Flag-tagged BpiP, *in trans*, transformed with **E**) plasmid pYC153. pYC153 was derived from pTM61 that constitutively express green fluorescent protein (GFP) under the control of the P*flaB* promoter. The construct also carries determinants for replication in both *E*. *coli* and *B*. *burgdorferi*, gentamicin resistance (*aacC1*) cassette, upstream regions of *bpiP* and *bpiP* fused to 3x- FLAG tag. Spirochetes were fractionated into outer membrane vesicle (OMV) and protoplasmic cylinder (PC) fractions as described in the Materials and Methods section. Fractions were separated on 12% SDS-PAGE gel stained with Coomassie brilliant blue (**A, C**) or transferred to PVDF membranes and subjected immunoblot analysis (**B, D**) using specific antisera indicated to the right of each blot. **(F).** Periplasmic and spheroplasmic fractions were separated on SDS-PAGE, transferred to PVDF membrane as described in the Materials and Methods section and subjected to immunoblot analysis with anti-FLAG tag, anti-Green Fluorescent Protein (gfp) or anti-FlaB antibodies. Blots were developed using goat-anti-mouse or anti-rabbit IgG conjugated with HRPO as secondary antibody and Enhanced Chemiluminescence. Lanes 1) Outer membrane vesicle fraction (OMV) 2) Periplasmic cylinder (PC). Note detection of BpiP or BpiP-FLAG only in the PC fraction using either anti-BpiP or anti-FLAG antibody. P66 localized to OMV and PotA to PC fraction in both wild type and *bpiP* mutant complemented with pYC153. Molecular masses in kDa are indicated to the left.

Since BpiP was annotated as an OmpA/C like protein, we determined the localization of BpiP by fractionating *Bb* into Outer-membrane vesicles (OMVs) and protoplasmic cylinder (PC) fractions. The periplasmic or spheroplasmic proteins of *Bb* were also analyzed for presence of BpiP. As shown in Fig 2B, immunoblot analysis revealed presence of BpiP in the PC fraction while a known outer membrane protein (P66) localized to the OMV fraction. Moreover, PotA, a known inner membrane protein, also localized to the PC fraction as expected. The total protein profiles of the OMV and PC fractions are shown in Fig 2A. The localization of the BpiP in the PC fraction was also determined by transforming *bpiP* mutant with a borrelial shuttle vector expressing BpiP under the control of its native promoter and fused to 3XFLAG-tag (Fig 2E). Immunoblot analysis using anti-FLAG-tag antibodies revealed the localization of BpiP fused to FLAG-tag in the PC fraction while P66 and PotA localized to the OMV and PC fractions respectively, as expected (Fig 2D). Protein profiles of the OMV and PC fractions of *bpiP* mt carrying pYC153 are shown in Fig 2C. Immunoblot analysis of *Bb* (*bpiP* mutant carrying pYC153), spheroplasmic and periplasmic proteins using anti-FLAG-Tag antibodies revealed the presence of BpiP in the periplast fraction although the amount of BpiP within the whole cells and spheroplasmic fractions were higher (Fig 2F, α-FLAG-Tag). As a control, FlaB was localized abundantly in the periplasmic fraction of *Bb* consistent with its localization within the periplasmic space of *Bb* (Fig 2F, α-FlaB). However, there was no detection of gfp in the periplast fraction suggesting that there is no contamination of the cytosolic proteins under the conditions used to prepare the periplasts and spheroplasts (Fig 2F, anti-gfp). These observations suggest that although BpiP (BB0167) was annotated as an Outer Membrane Protein A/C like protein, it is localized in the PC fraction and is likely to accumulate or be detected in the periplasm of *Bb*.

### BpiP binds to borrelial PG

**Fig 3:**
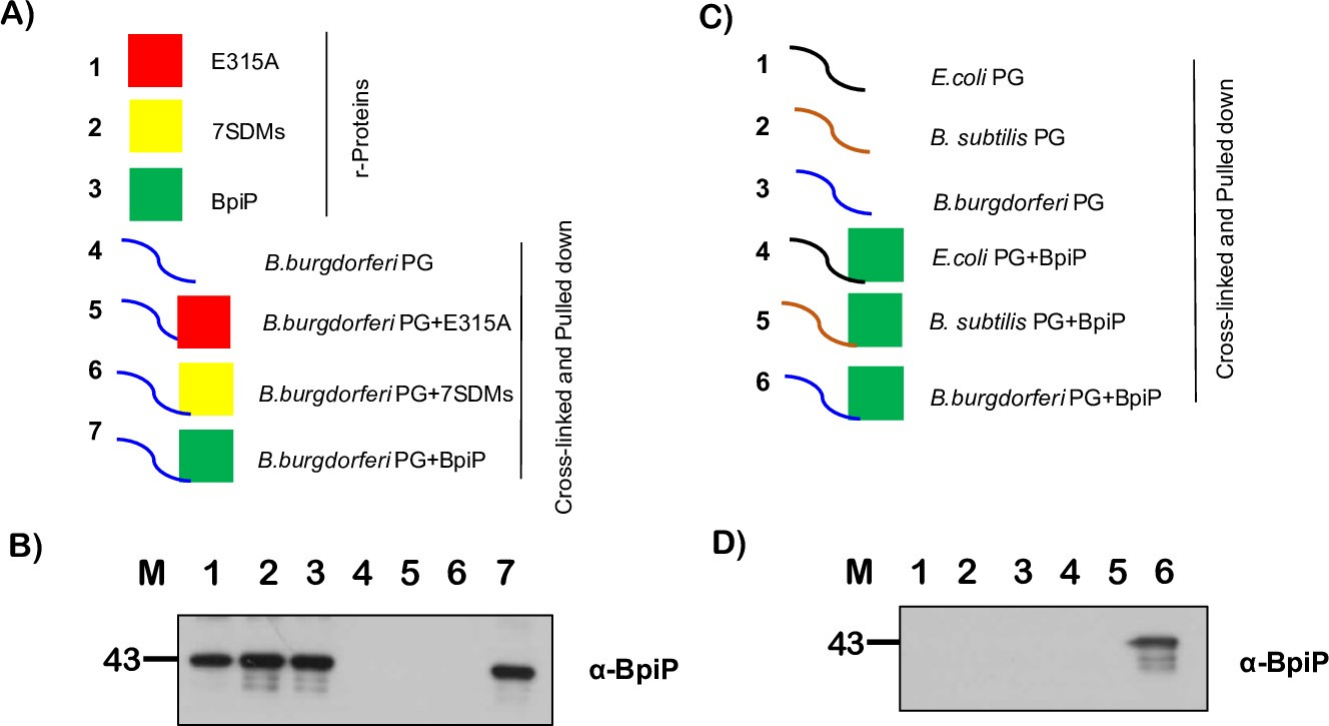
Interactions of BpiP with PG. **(A)** Schematic representation of recombinant BpiP proteins with site-specific changes at (1) position E315 or (2) with 7 site directed mutations (7SDMs) replacing amino acids of PG-interacting motif of BpiP with alanines or glycine, (3) wild type BpiP, (4) borrelial PG and (5–7) cross-linked complexes of various recombinant BpiP proteins with borrelial PG that were subjected immunoblot analysis. **(B)** Recombinant BpiP proteins (Lane 1–3), purified borrelial PG (Lane 4) and complexes of recombinant BpiP proteins crosslinked with borrelial PG were precipitated (pulled down) by centrifugation, dissociated via boiling and supernatants were separated as described in the flow chart in S3 Fig (Lanes 5–7) on 12% SDS-PAGE gel, transferred to PVDF membranes. Immunoblot analysis was performed using anti-BpiP serum followed by goat-anti-mouse IgG conjugated with HRPO and blots developed using Enhanced Chemiluminescence. Lanes 1) rE315ABpiP, 2) r7SDM BpiP 3) rBpiP, 4) PG only, 5) rE315A-BpiP cross-linked to PG, 6) r7SDM cross-linked to PG, 7) rBpiP cross-linked to PG. Molecular masses (M) in kilo Daltons are shown to the left. **(C)** Schematic representation of PG from *E*. *coli*, *B*. *subtilis*, *B*. *burgdorferi* either alone or cross-linked with BpiP and pulled down by centrifugation as complexes. Samples were subjected to boiling and supernatants were separated on 12% SDS-PAGE gel, transferred to PVDF membrane and detected for the presence of BpiP by immunoblot analysis **(D)** Immunoblot analysis was performed using anti-BpiP serum followed by goat-anti-mouse IgG conjugated with HRPO and blots developed using Enhanced Chemiluminescence. Lane 1) *E*. *coli* PG alone, 2) *B*. *subtilis* PG alone, 3) *B*. *burgdorferi* PG alone, 4) BpiP cross-linked to *E*.*coli* PG, 5) BpiP cross-linked to *B*.*subtilis* PG, 6) BpiP cross-linked to *Bb* PG. Molecular masses (M) in kilo Daltons are shown to the left.

Recombinant BpiP (rBpiP) with N-terminal Maltose Binding Protein (MBP) was purified and MBP was cleaved using TEV protease (S1 Fig). Interactions of BpiP with purified borrelial PG were analyzed after cross-linking with the reagent DTSSP as described in the Materials and Methods (Figs S3 and 3A). BpiP binding to PG was detected using anti BpiP serum generated against C-terminal region of BpiP purified using 7X His-tag [30]. The full-length wild-type BpiP bound borrelial PG (Fig 3B, Lane 7). However, BpiP with a site-specific change at E315A (Fig 3B, Lane 5) or BpiP with 7 conserved residues of the PG-binding motif (shown in red in Fig 1A) replaced with alanines or glycine showed no binding to borrelial PG (Fig 3B, Lane 6). As expected, purified PG by itself did not react with BpiP antibodies (Fig 3B, Lane 4) and the levels of wild type BpiP, E315A and 7SDMs used in the binding studies were similar as shown by their reactivity to anti BpiP antibodies (Fig 3B, Lanes 1–3). This biochemical analysis demonstrates the binding of BpiP to borrelial PG via its PG-binding motif. In addition, we also determined if BpiP interacted with heterologous PG from *E*. *coli* and *B*. *subtilis* that have *meso*-DAP or L-Lysine as the third amino acid in their pentapeptide crosslinks, respectively, using the procedure described for BpiP interaction with borrelial PG (Fig 3C). Immunoblot analysis using anti-BpiP serum revealed interaction of BpiP only with borrelial PG (Fig 3D, Lane 6) but not with *E*. *coli* or *B*. *subtilis* PG (Fig 3D, Lanes 4 and 5) or with any of the purified PGs (Fig 3D, Lanes 1–3). These observations demonstrated that BpiP interacted with borrelial PG and that the nature of the composition of the pentapeptide crosslink is critical for this interaction.

### *bpiP* is transcriptionally up-regulated under *in vitro* growth conditions mimicking midgut of fed ticks

**Fig 4:**
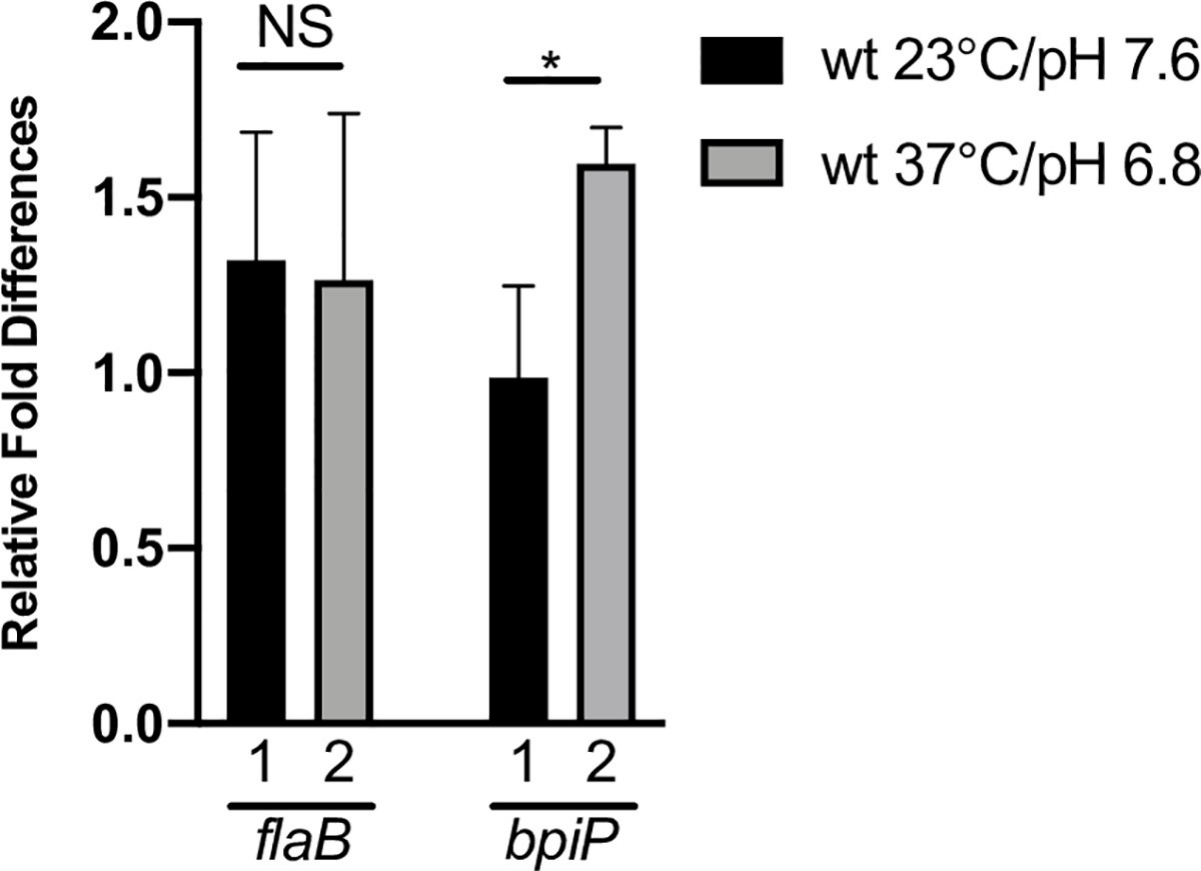
Transcriptional analysis of *bpiP*. **(A)** Total RNA from wt grown at pH7.6/23°C or pH6.8/37°C was converted to cDNA and quantitative real-time PCR was performed as described in the Materials and Methods. The value for each sample was normalized to the value of recA and change in the C_*t*_ value for *flaB* or *bpiP* shown is an average of three replicates. The ΔΔC_*t*_ values are shown as fold difference. The asterisk indicates the levels of significance as follows: *p<0.05.

Shifting cultures from conditions mimicking midgut of unfed (pH7.6/23°C) to that of fed ticks (pH6.8/37°C) has been shown to drive significant alterations in gene expression in response to changes in the environmental signals [37–39]. Previous studies have shown that the transcriptional levels of *bpiP* were elevated in fed infected nymphs [36]. As shown in Fig 4, the transcriptional levels of *bpiP* were significantly higher in *Bb* grown under *in vitro* conditions mimicking the fed ticks (pH6.8/37°C) compared to that grown under unfed tick condition (pH 7.6/23°C). It should be pointed out that the *in vitro* growth conditions mimicking fed ticks do not completely reflect the *in vivo* conditions but nonetheless increased transcriptional levels of *bpiP* under fed-tick conditions is suggestive of the key environmental cues that alter its levels of expression.

### Generation and *in vitro* growth phenotype of *bpiP* mutant and complemented strains

**Fig 5:**
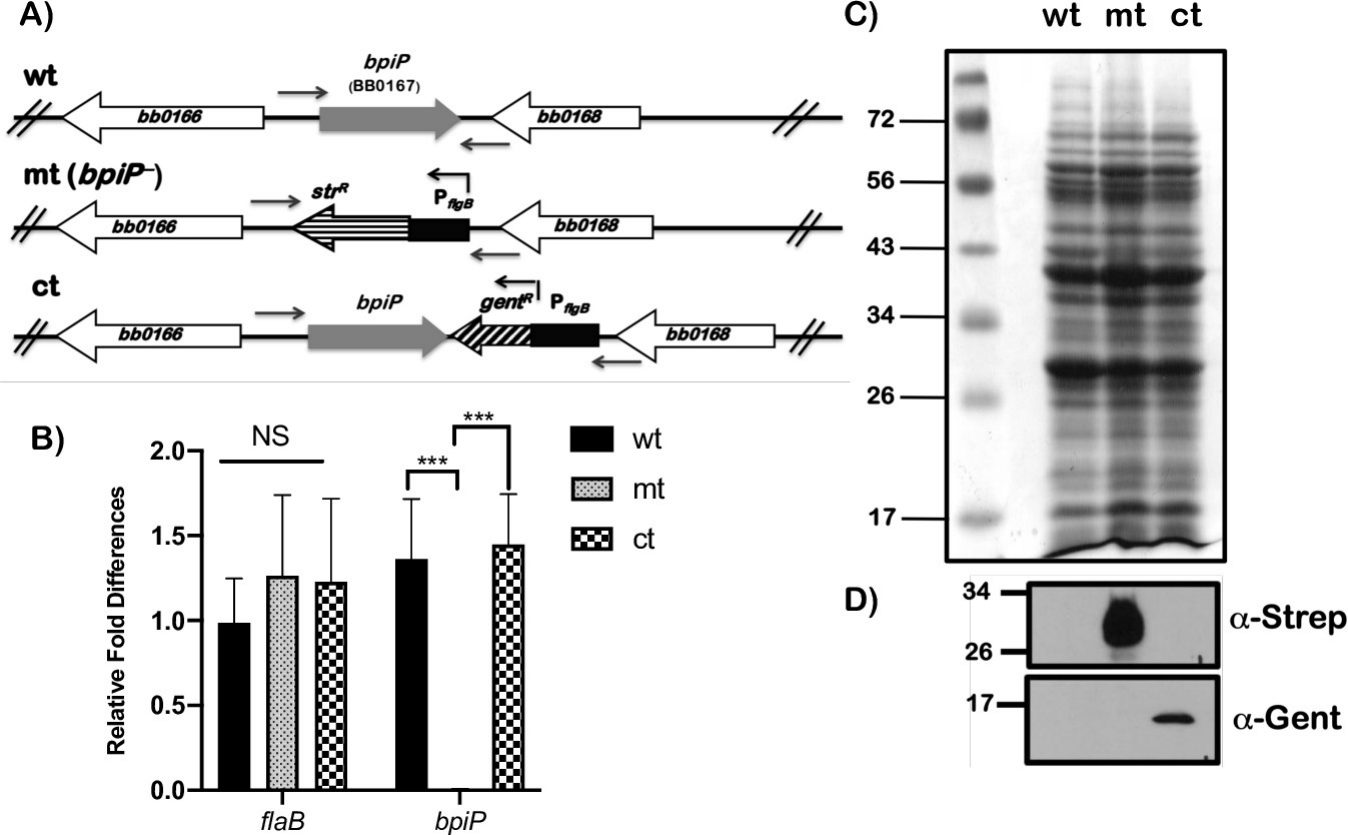
Generation of the *bpiP* mutant and complement strains. **(A)** Schematic representation of the genetic manipulation performed to generate *bpiP* (*bb_0167*) mutant and cis-complemented strains. **(B)** Total RNA from wt, mt and ct grown at pH7.6/32°C was converted to cDNA and quantitative real-time PCR was performed as described in the Materials and Methods section. The levels of *bpiP* transcripts were absent as expected in the mt compared the control strains. The change in the C_*t*_ value for each sample was subjected to an unpaired *t* test in GraphPad Prism software. The asterisk indicates the levels of significance as follows: *** *p*<0.001. Total protein lysates from wt (Lane 1), mt (Lane 2) and ct (Lane 3) were separated on 12% SDS-PAGE gel and **(C)** stained with Coomassie blue or **(D)** transferred to PVDF membrane for immunoblot analysis using antibodies specific to streptomycin resistance (α-Strep, mt) or gentamicin resistance (α-Gent, ct) proteins synthesized due to the expression of *aadA* and *gentR* resistance genes under the control of borrelial promoter P_*flgB*_.

In order to determine the role of BpiP in the patho-physiology of *Bb*, we undertook a genetic approach by deleting *bpiP* in *Bb* strain B31-A3 that has the full complement of infection-associated plasmids [40–43]. As shown schematically in Fig 5A, we replaced *bpiP* with streptomycin resistance gene (*aadA*) under the control of a borrelial promoter P_*flgB*_ and selected mutants in the presence of streptomycin (50 μg/ml). A cis-complemented strain was generated by restoring an intact copy of *bpiP* with gentamicin resistance gene (GentR) under the control of P_*flgB*_ (Fig 5A, ct) and selected in the presence of gentamicin (50 μg/ml). The deletion and cis-complemented strains were confirmed by PCR using primers flanking *bpiP* as shown schematically. There was no transcription of *bpiP* in the mutant strain compared wt or ct strains while the levels of *flaB* were not statistically different between all three strains (Fig 5B). The total protein lysates from each of these strains resolved by SDS-12.5% PAGE and stained with Coomassie Blue served as a loading control (Fig 5C). Antiserum generated against antibiotic resistance proteins (recombinant streptomycin-resistance and gentamicin-resistance proteins) was used to determine the synthesis of streptomycin resistance protein in the *bpiP* mutant and gentamicin resistance protein in the cis-complemented strain, respectively, while the parental strain showed no reactivity to either antisera as expected (Fig 5D). We also confirmed the deletion and restoration of an intact copy of *bpiP* by whole genome sequencing of DNA from wt, mt and ct strains [41]. Moreover, sequence analysis of the genomic DNA also revealed that the plasmid profile was identical between wt, mt and ct strains. There were no significant differences in the growth rates of wt, mt and ct strains when propagated under *in vitro* conditions reflecting growth in laboratory (pH7.6/32°C); un-fed tick midgut (pH7.6/23°C); or fed tick midgut (pH6.8/37°C; S4 Fig). A trans-complemented strain was generated by transforming the *bpiP* mutant using a borrelial shuttle vector pYC153 obtained by cloning 1kb upstream of *bpiP* and *bpiP-*3XFLAG-tag at the Kpn1 site present in the plasmid pTM61 (Fig 2E) [44]. The trans-complemented strain synthesized BpiP that was localized to the protoplasmic cylinder (PC) fraction, which was detected by immunoblot analysis using anti-FLAG-tag antibodies (Fig 2D).

### Synthesis of select borrelial proteins in *bpiP* mutant

**Fig 6:**
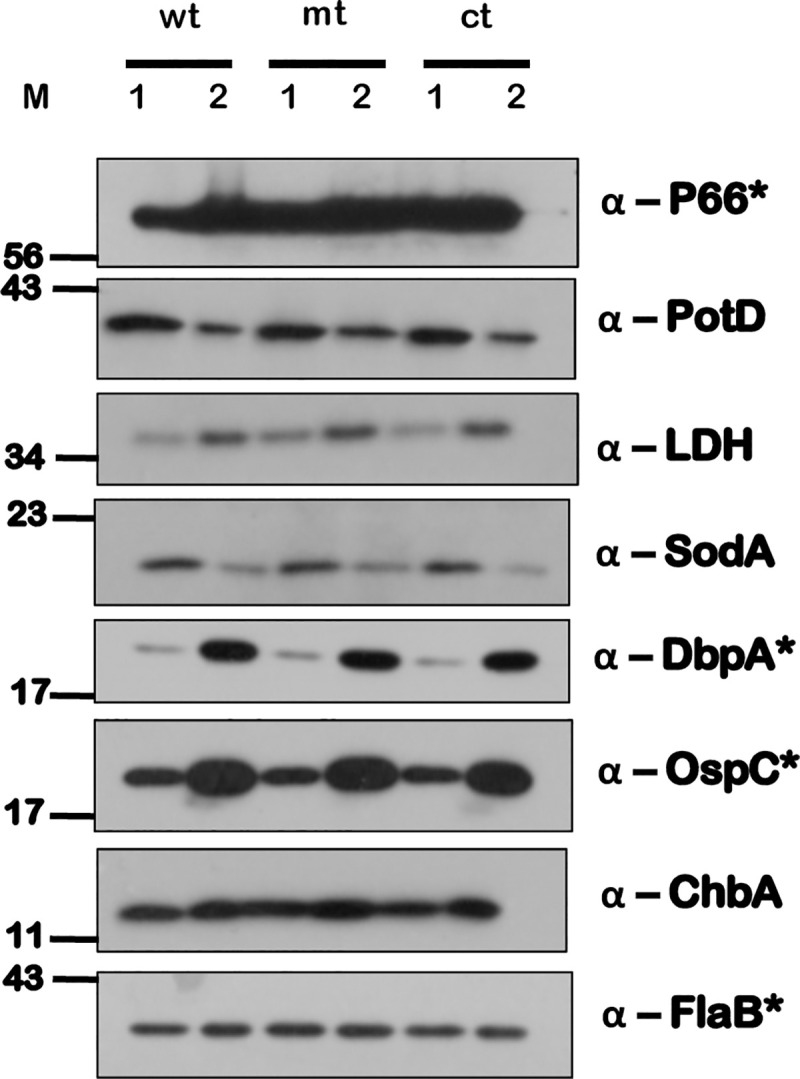
Levels of pathogenesis-related proteins in *bpiP* mutant. Total protein lysates of wt, mt, ct strains grown under conditions mimicking midgut of unfed tick (Lane 1, pH 7.6/ 23°C) and shifted to conditions mimicking midgut of fed tick (Lane 2, pH 6.8/37°C) were separated on a 12% SDS-PAGE gel, transferred to PVDF membrane and subjected to immunoblot analysis using antisera against various borrelial proteins indicated to the right of each blot. Goat anti-mouse HRPO-conjugated antibodies were used as secondary antibodies and blots were developed using ECL system. Molecular masses in kilo Daltons (kDa) are indicated to the left. (*indicate protein samples diluted 1:10 due to quality of the antibody reagent).

In order to determine if the deletion of *bpiP* resulted in changes in key borrelial proteins, we analyzed whole cell protein lysates of wt, mt and ct strains propagated under *in vitro* conditions either mimicking the midgut of unfed (Fig 6, Lane 1 pH7.6/23°C) or fed (Fig 6, Lane 2 pH6.8/37°C) tick by immunoblot analysis using specific antisera. Consistent with several previous reports, the levels of Outer surface protein C (OspC), Decorin binding protein A (DbpA) and Lactate dehydrogenase (LDH) were elevated under fed tick conditions compared to the unfed tick condition [19, 38, 40, 45]. Levels of PotD and SodA were elevated at the unfed tick condition while levels of several proteins such as P66, ChbA and FlaB were similar under both conditions serving as controls for equal loading of proteins. The levels of the aforementioned borrelial proteins were similar in wt, mt and ct indicating that the deletion of *bpiP* did not have any significant impact on the levels of several key pathogenesis-related lipoproteins and select enzymes involved in metabolic and oxidative stress response pathways.

### *bpiP* mutant is attenuated for infection in C3H/HeN mice

**Fig 7:**
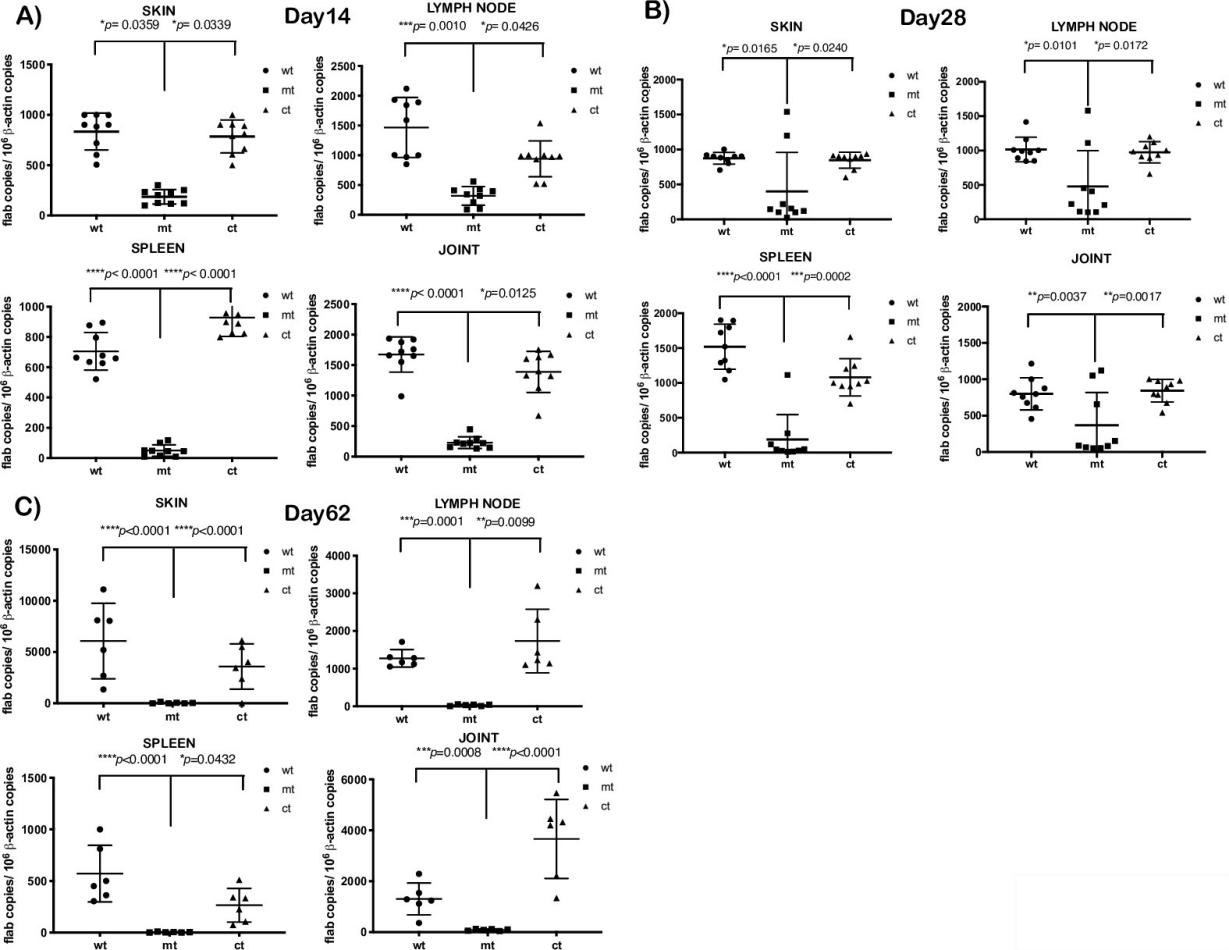
Quantitative real-time PCR analysis of spirochetal burden in C3H/HeN mice infected with *bpiP* mutant. Groups (*n* = 3) of 4-week old C3H/HeN mice were intradermally infected via needle inoculation with wt, mt and ct strains with 10^5^ spirochetes per mouse and data from 3 such independent experiments (total *n* = 9 mice) were analyzed. Total genomic DNA was isolated from tissue (skin, lymph node, spleen and one joint) using the High Pure PCR template preparation kit and was then subjected to quantitative real-time PCR. Numbers of borrelial *flaB* copies were normalized against total mouse β-actin. Data analyzed using a one-way analysis of variance test with Tukey’s multiple-comparison test. Statistical significance was accepted when *p* values were less than 0.05. Tissues samples analyzed were collected at day **(A)** 14, **(B)** 28 and **(C)** 62 post-infection.

We challenged C3H/HeN mice with 1x10^5^ wt, mt or ct strains via needle inoculation and determined the levels of infectivity at day 14, 28 (*n* = 3; three independent experiments) or 62 (*n* = 3, two independent experiments) post-infection and select tissues were isolated aseptically as indicated in Table 1. Isolated tissues were inoculated into BSKII medium and scored for growth after a single blind pass at day 5 by examining cultures using dark field microscopy at day 15 post-isolation in primary and blind-pass cultures. As shown in Table 1, the mt strain was isolated from different tissues in 8 out of 9 mice at day 14 while only 4 out of the 9 mice supported growth of the mt at day 28. But at day 62, none of the C3H/HeN mice (6 out of 6) were infected with the mt strain demonstrating a significant survival defect during long-term infection. This is in contrast to all mice being infected with wt and ct strains at day 14, 28 and 62 post-infection. We further enumerated the number of spirochetes in select tissues (skin, one lymph node, spleen and one joint) by quantitative real time PCR (Fig 7). While it was possible to recover mt spirochetes from infected tissues in 8 out 9 mice at day 14, qPCR analysis revealed a significant reduction in the *Bb* burden in all tissues examined such as skin, lymph node, spleen and one of the joints compared to the wt and ct strains (Fig 7A). A significant reduction in *Bb* burden in mice infected with mt at day 28 in the tissues examined compared to the control strains (Fig 7B) while tissues from all mice infected with mt did not exhibit detectable pathogen burden by qPCR at day 62 (Fig 7C). These infectivity studies clearly demonstrated that BpiP plays a role in the survival of *Bb* in C3H/HeN mouse model of Lyme disease.

**Table 1:** Analysis of infectivity in C3H/HeN mice with wt, *bpiP* mt and *bpiP* ct strains at day 14, 28 and 62 post infection.

Strains	No. of cultures positive/total number.						Total number infected
Day 14	Skin	Lymph Node	Spleen	Heart	Bladder	Joint	
wt	9/9	9/9	9/9	9/9	9/9	9/9	9/9
mt	8/9	8/9	3/9	5/9	3/9	6/9	8/9
ct	9/9	9/9	9/9	9/9	9/9	9/9	9/9
Day 28							
wt	9/9	9/9	9/9	9/9	9/9	9/9	9/9
mt	2/9	3/9	2/9	4/9	4/9	3/9	4/9
ct	9/9	9/9	9/9	9/9	9/9	9/9	9/9
Day 62							
wt	6/6	6/6	6/6	6/6	6/6	5/6	6/6
mt	0/6	0/6	0/6	0/6	0/6	0/6	0/6
ct	6/6	6/6	6/6	6/6	6/6	6/6	6/6

### Infectivity analysis of *bpiP* mutant in immunodeficient SCID mice

**Fig 8:**
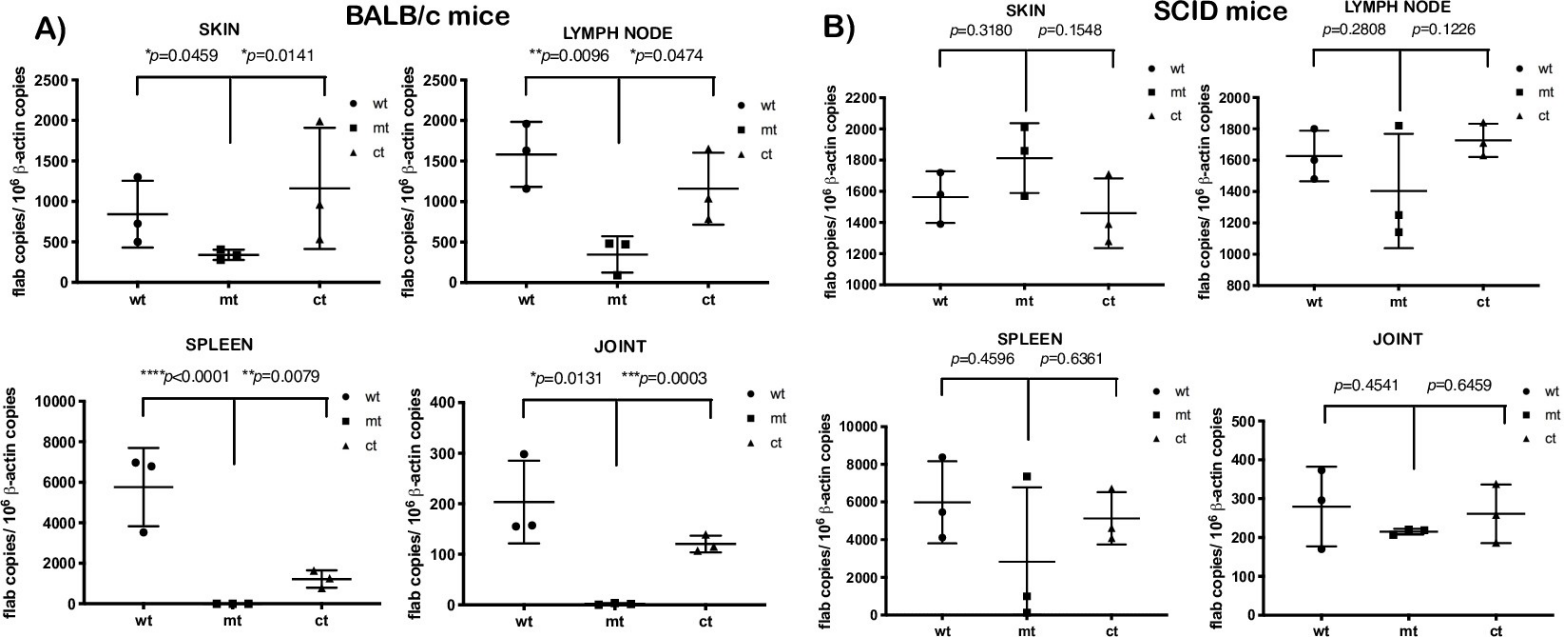
Quantitative real-time PCR analysis of spirochetal burden in BALB/c or SCID mice infected with *bpiP* mutant. Groups (*n* = 3) of 4-week old immunocompetent BALB/c or immunodeficient SCID mice were intradermally infected via needle inoculation with wt, mt and ct strains with 10^5^ spirochetes per mouse and were analyzed. Total genomic DNA was isolated from tissue (skin, lymph node, spleen and one joint) using the High Pure PCR template preparation kit and was then subjected to quantitative real-time PCR. Numbers of borrelial *flaB* copies were normalized against total mouse β-actin. Data analyzed using a one-way analysis of variance test with Tukey’s multiple-comparison test. Statistical significance was accepted when *p* values were less than 0.05. Tissues samples analyzed were collected at day 28 from **(A)** BALB/c **(B)** SCID mice.

To determine if the induction of the adaptive immune response of the host contributes to the reduced infectivity of mt spirochetes at day 28 and 62, we compared the levels of infection of the mt in immunocompetent BALB/c and immunodeficient SCID mice. As shown in Table 2, no viable mt spirochetes were detected following infection of BALB/c mice at day 28 while viable spirochetes were isolated from all tissues tested from SCID mice. In addition, as shown in Fig 8, relative spirochetal burden from select tissues by qPCR revealed a significant reduction in *Bb* burden in tissues from BALB/c mice (Fig 8A) while the mt spirochetes were readily detectable in tissues from the SCID mice (Fig 8B). These observations suggest the role of the adaptive immune response of the host contributes to the *in vivo* phenotype of the *bpiP* mutant.

**Table 2:** Analysis of infectivity of immunocompetent BALB/c mice and immunodeficient SCID mice with wt, mt and ct strains at day 28 post infection.

Strains	No. of cultures positive/total number.						Total number infected
BALB/c mice	Skin	Lymph Node	Spleen	Heart	Bladder	Joint	
wt	3/3	3/3	3/3	3/3	3/3	3/3	3/3
mt	0/3	0/3	0/3	0/3	0/3	0/3	0/3
ct	3/3	3/3	3/3	3/3	3/3	3/3	3/3
SCID mice							
wt	3/3	3/3	3/3	3/3	3/3	3/3	3/3
mt	3/3	3/3	3/3	3/3	3/3	3/3	3/3
ct	3/3	3/3	3/3	3/3	3/3	3/3	3/3

### *bpiP* mutant is attenuated for survival in the mouse-tick mouse cycle of *Bb* infection

**Fig 9:**
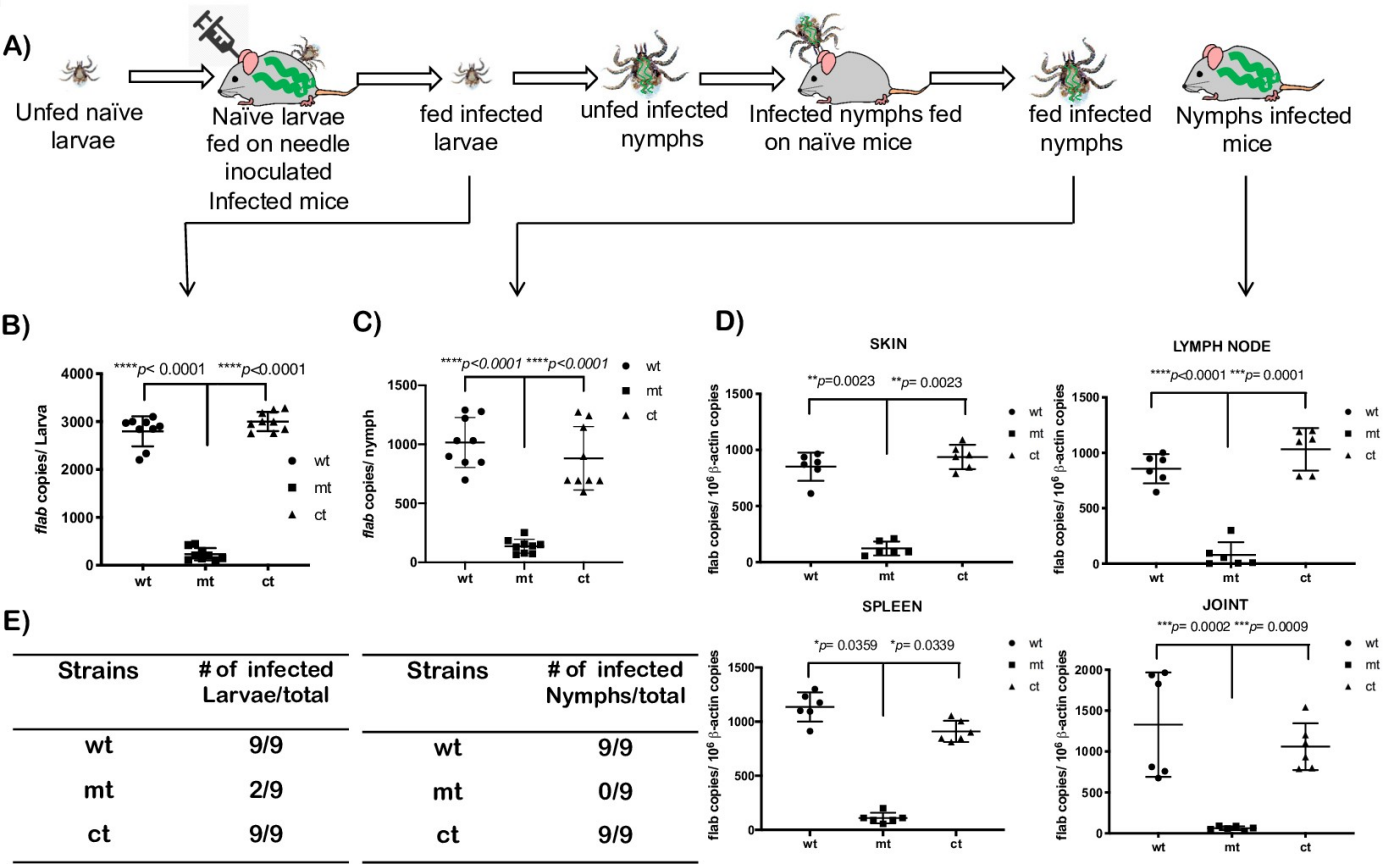
Quantitative real-time PCR analysis of the spirochetal burden in mice challenged with the *bpiP* mutant via *I*. *scapularis* nymphs. **(A)** Schematic representation of the experimental plan used in mouse-tick-mouse cycle of *Bb* infection. Naïve *I*. *scapularis* larvae were fed to repletion on C3H/HeN mice infected with wt, mt or ct strains with 10^5^ spirochetes per mouse via needle inoculation. *Bb* burden in a proportion of fed larvae (3 per mouse) determined by **(B)** qPCR or **(E)** examined for the presence of spirochetes by dark field microscopy. Another proportion of fed larvae were allowed to molt to nymphs in an incubator maintained at 22°C with 90% humidity and a 15-hour light /9-hour dark cycle. Groups (*n* = 3) of 4-week-old naïve C3H/HeN female mice were challenged with 10 infected nymphs and after nymphs fed to repletion. *Bb* burden was determined from 3 nymphs per mouse by (**C**) qPCR or (**E**) examined for the presence of spirochetes in the midgut by dark field microscopy Infectivity analysis was done at day 14 post-infection by propagating several tissues for the presence of spirochetes (Table 3). A portion of spleen, one lymph node, spleen and one joint was used to extract total genomic DNA and **(D)** *Bb* burden was determined quantitative real time PCR. The infectivity analysis was done twice and results of both the experiments are reported together for mice infected with nymphs. Numbers of borrelial *flaB* copies were normalized against total mouse β-actin copies. The differences between the number of wt and mt or mt and ct in mouse tissues were statistically analyzed using a one-way analysis of variance test with Tukey’s multiple-comparison test. Statistical significance was accepted when *p* values were less than 0.05.

We then determined if the attenuated infectivity phenotype observed in the mammalian host following needle challenge could be reproduced using a tick infection model. The mouse-tick-mouse cycle of infection was performed as shown schematically in Fig 9A. We challenged naive C3H/HeN mice with wt, mt and ct strains via intradermal needle inoculation with 10^5^ spirochetes per mouse and allowed *I*. *scapularis* larvae to feed to repletion on infected mice at day 5 post-challenge. Three fed larvae from each mouse (total 9 for each strain) were processed for qPCR to determine the spirochetal burden in fed larvae (Fig 9B) and three fed larvae from each mouse (total 9 for each strain) were examined for presence of spirochetes (Fig 9E). The rest of the fed larvae were allowed to molt to nymphs in an incubator as described in Materials and Methods section. As shown in Fig 9B, there was a significant reduction in number of spirochetes in larvae fed on mice infected with mt compared to that from mice infected with wt and ct strains. We then allowed 10 infected nymphs to feed to repletion on naïve C3H/HeN (*n* = 3, two biological replicates, data from both experiments combined in Table 3 and Fig 9) and examined 3 fed nymphs from each mouse (total 9) infected with wt, mt or ct strains for the presence of spirochetes by qPCR (Fig 9C) or by dark field microscopy (Fig 9E). As shown in Fig 9C, there was a significant reduction in the number of spirochetes in fed nymphs infected with mt compared to nymphs infected with wt or ct strains. The levels of transmission of wt, mt or ct to naive C3H/HeN mice following challenge with infected nymphs were determined by isolating different tissues aseptically for isolation of viable spirochetes in BSKII borrelial growth medium (Table 3) or by qPCR for spirochete burden (Fig 9D) at day 14 post-tick challenge. Mutant spirochetes were isolated from the skin and lymph nodes of 1 out 6 C3H/HeN mice challenged with infected nymphs while there were no viable spirochetes in other tissues examined. This is in contrast to mice challenged with nymphs infected with wt or ct strains where every tissue was positive for viable spirochetes. Moreover, these mice had significantly higher *Bb* burden compared to mice challenged with nymphs infected with the mutant. These studies demonstrated that *bpiP* mt was acquired in lower numbers by larvae from infected mice leading to reduced transmission by infected nymphs to naive C3H/HeN mice.

**Table 3:** Analysis of infectivity of C3H/HeN mice challenged with wt, mt and ct strains via infected *Ixodes scapularis* nymphs.

Strains	No. of cultures positive/total number						Total number infected
	Skin	Lymph Node	Spleen	Heart	Bladder	Joint	
wt	6/6	6/6	6/6	6/6	6/6	6/6	6/6
mt	1/6	1/6	0/6	0/6	0/6	0/6	1/6
ct	6/6	6/6	6/6	6/6	6/6	6/6	6/6

### Sensitivity of *bpiP* mutant to vancomycin

**Fig 10:**
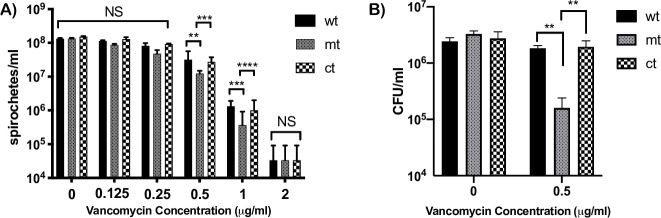
Sensitivity of *bpiP* mutant to vancomycin. All three strains (wt, mt and ct) were propagated at 10^6^/ml in BSKII growth medium with (A) 0, 0.125, 0.25, 0.5, 1 and 2 μg/ml of Vancomycin, and grown at pH7.6/32°C with 1% CO_2_. **(A)** Spirochetes were enumerated every 24 hrs by dark-field microscopy and the number of spirochetes/ml at day 5 post-treatment is shown. (**B**) Colony forming units of cultures with 0 or 0.5 μg/ml of vancomycin at day 5 post-treatment were determined by diluting cultures of all three strains to 1:100,000 in 1X BSKII medium, plated on BSKII agar overlays and the final CFU/ml of the treated cultures were calculated after 10 days of incubation at 32°C with 1% CO_2_. The CFUs were determined in triplicate, one of three independent experiment is shown. Statistically analysis was done using unpaired *t*-test. Statistical significance was accepted when *p* values were less than 0.05. The asterisks indicate the levels of significance as follows: ****, *p*<0.0001; ***, *p*<0.001; **, *p*<0.01.

We then investigated the mechanistic basis for lack of infectivity in mouse models of infection and to connect the functional contribution of BpiP via its interactions with borrelial peptidoglycan to infectivity of *Bb* in mouse/tick models of LD. We performed a series of analyses to determine the role of BpiP in the ability of spirochetes to withstand exogenous or endogenous anti-bacterial effector molecules that specifically cause stress to the bacterial cell wall/cell envelope. Vancomycin, a glycopeptide antibiotic that inhibits PG synthesis by non-covalent binding to D-alanyl-D-alanine (D-ala-D-ala) terminus of the pentapeptide cell wall precursor displays activity against *Bb* under *in vitro* and *in vivo* growth conditions and has been shown to reduce the cell wall stiffness and motility of *Bb* [46, 47] As shown in Fig 10A, 0.5 and 1.0 μg /ml vancomycin resulted in a significant reduction in the growth of mt compared to wt or ct strains. Concentrations lower than 0.5 or greater than 1.0 μg /ml did not result in significant differences in the growth rates between mt and the control strains. Colony forming units (cfus) of cultures with 0 or 0.5 μg/ml of vancomycin at day 5 post-treatment were obtained by plating on BSKII agar overlays. The mt exhibited a significant reduction in the number of cfu/ml compared to wt or ct strain following treatment with 0.5 μg/ml of vancomycin (Fig 10B).

### Sensitivity of *bpiP* mutant to increased osmotic stress

**Fig 11:**
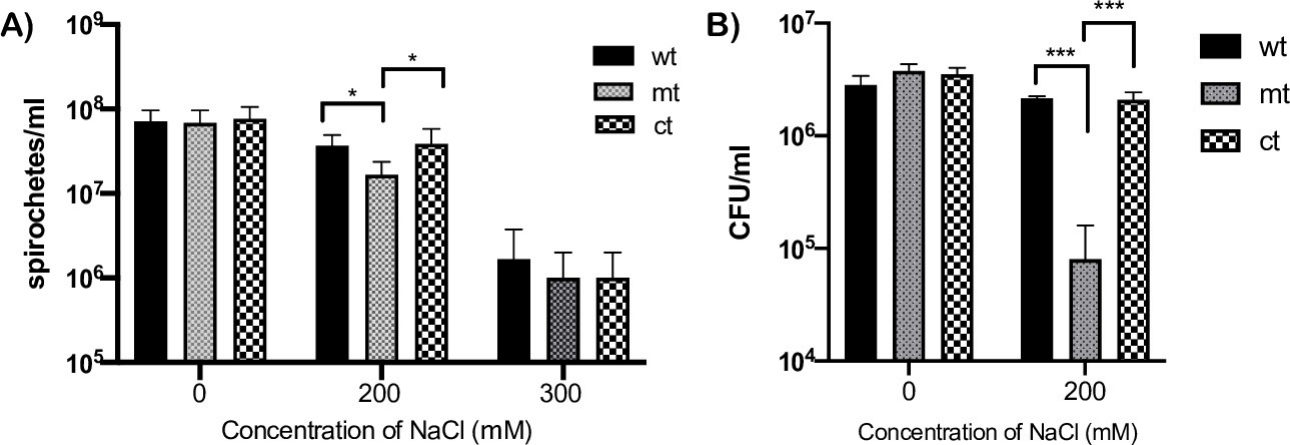
Sensitivity of *bpiP* mutant to osmotic stress. All three strains (wt, mt or ct) were seeded at a density of 1x10^6^ spirochetes per ml in 5 ml of normal BSKII or BSKII supplemented with 200 or 300 mM of sodium chloride. **(A)** Cultures were incubated at 32°C, enumerated every 24 hours and number of spirochetes/ml at 5 days post-treatment is shown. **(B)** Colony forming units of cultures with 0 or 200 mM concentration of sodium chloride at day 5 post-treatment were determined by diluting cultures of all three strains to 1:100,000 in 1X BSKII medium, plated on BSKII agar overlays and the final CFU/ml of the treated cultures were calculated after 10 days of incubation at 32°C with 1% CO_2_. Data shown are means of three technical replicates with error bars representing Standard Error Mean and is representative of one of the three experiments. Statistical analysis was done using unpaired *t*-test and the asterisks indicate levels of significance as follows: ***, p<0.001; *, *p* <0.05.

*bpiP* mutant had a lower growth rate in the presence of 200 mM of sodium chloride above the levels normally present in the BSKII borrelial growth medium after 120 hrs of growth (Fig 11A). The *bpiP* mutant had significantly lower cfu/ml with 200 mM concentration of sodium chloride compared to wt or mt or when grown with no additional supplementation of NaCl (Fig 11B). These observations suggest that lack of BpiP decreases the ability of Lyme spirochetes to withstand select physiological stressors.

### Sensitivity of *bpiP* mutant to lysosomal extracts

**Fig 12:**
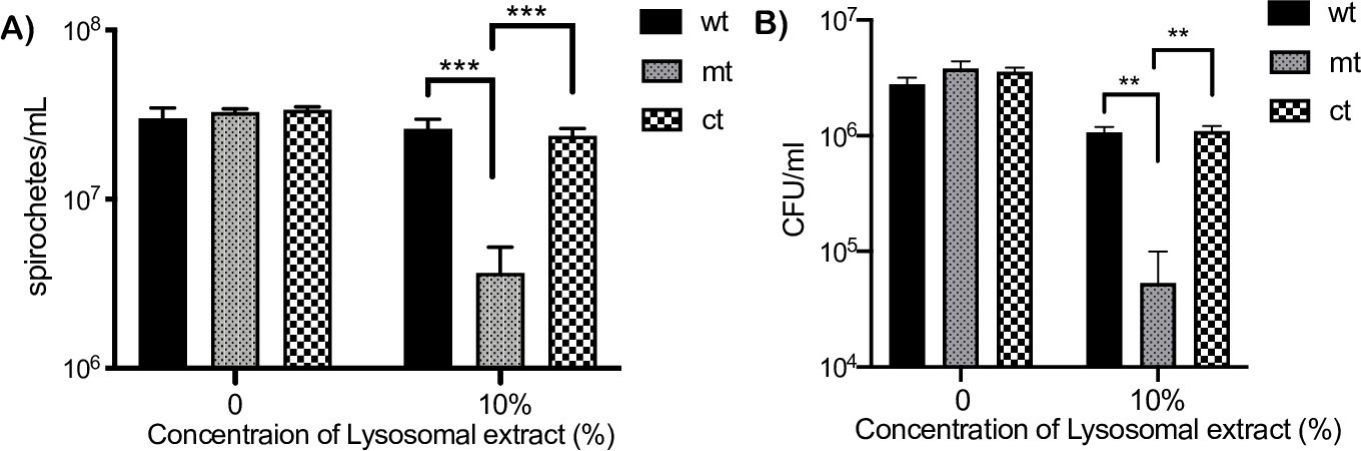
Sensitivity of *bpiP* mt to lysosomal extracts. All three strains (wt, mt, ct) were seeded at a density of 1×10^6^ spirochetes in 1ml BSKII media with or without 10% lysosomal extract obtained from murine bone marrow derived dendritic cells as described in the Materials and Methods section and incubated at 37°C, 1% CO_2_ for five days. **(A)** Spirochetes were enumerated every 24 hours and data shown is for the number of spirochetes/ml after 5 days of treatment. (**B**) Colony forming units of cultures with 0 or 10% lysosomal extract at day 5 post-treatment were determined by diluting cultures of all three strains to 1:100,000 in 1X BSKII medium, plated on BSKII agar overlays and the final CFU/ml of the treated cultures were calculated after 10 days of incubation at 32°C with 1% CO_2_. One of two independent experiments is shown. Statistical analysis of differences in the number of spirochetes between treated and untreated cultures were determined by unpaired *t* test. The asterisks indicate levels of significance as follows: ***, *p* <0.001; **, *p* <0.01.

We proceeded to determine if host-derived stressors that targeted the cell wall/cell envelope would also lead to a readily observable phenotype in the *bpiP* mutant. Numerous host-derived antibacterial effectors play a role in controlling bacterial infections. Many of the effectors impact the cell envelope and PG of bacterial cells. Since the *bpiP* had a significant colonization deficit within 14 days, post-infection, in immunocompetent mouse models, we determined the effects of lysosomal extracts from bone-marrow derived dendritic cells from C3H/HeN or C57BL6 mice. As shown in Fig 12A, *bpiP* mt incubated with lysosomal extract had a significant growth defect compared to the wt and ct strain. Moreover, the *bpiP* mutant also had significantly lower cfu/ml when exposed to 10% lysosomal extract compared to wt or mt or when grown without lysosomal extract (Fig 12B).

### Sensitivity of *bpiP* mutant to human or murine antimicrobial peptides, LL37 or mCRAMP

**Fig 13:**
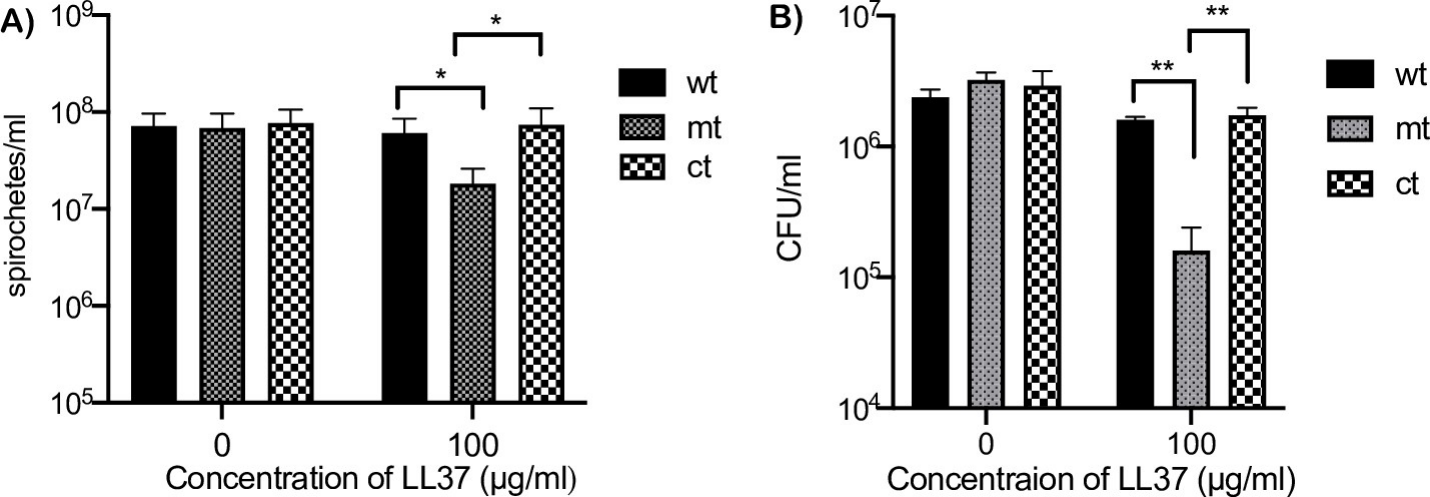
Sensitivity of *bpiP* mt to human antimicrobial peptides. All three strains (wt, mt and ct) were propagated at 10^5^/ml in BSKII growth medium at pH 6.8/32°C with 0 or 100 μg/ml LL37. **(A)** Spirochetes were enumerated every 24 hours and data shown is for the number of spirochetes/ml after 5 days of treatment. (**B**) Colony forming units of cultures with 0 or 100 μg/ml of LL37 were determined by diluting cultures of all three strains to 1:100,000 in 1X BSKII medium, plated on BSKII agar overlays and the final CFU/ml of the treated cultures were calculated after 10 days of incubation at 32°C with 1% CO_2_. The cultures were grown in triplicate and one out of two independent experiments is shown. Statistical analysis of the difference in the number of wt and mt or ct and mt spirochetes was done by unpaired *t* test. The asterisks indicate levels of significance as follows: **, *p*<0.01; *, *p*<0.05.

We then tested the phenotype of the mt in the presence of host-derived antimicrobial peptides from rodents and humans. DC lysosomal extract contains many different antimicrobial effectors [48]. Among these, antimicrobial peptides may play a role in reducing the infectivity of *bpiP* mutant in the mammalian host. Moreover, during skin infections, cathelicidin-type antimicrobial peptides are induced [49]. The cathelicidin α-helical peptides such as LL37 can rapidly permeabilize the cell membrane of bacteria [50]. Thus, we tested the sensitivity of wt, mt and ct strains with 100 μg/ml of human and mouse cathelicidins, LL37 and mCRAMP, respectively, in BSKII medium at pH6.8 /37°C and observed that mt was sensitive to human cathelicidin (Fig 13A) while mCRAMP had no inhibitory effect on mt compared to the control strains (S2 Fig). In addition, the mutant had significantly lower cfu/ml in the presence of 100 μg/ml of LL37 compared wt or ct strains while there were no significant differences in the cfu/ml of these strains in the absence of LL37 (Fig 13B). It is possible the effects of these anti-microbial peptides require environments that are more acidic than that conferred with BSKII medium at pH6.8, although lower pH as such has a higher growth inhibitory effect on Lyme spirochetes. These observations suggest that BpiP contributes to the integrity of the cell wall/cell envelope against multiple antimicrobial effectors that could synergistically increase the sensitivity of *bpiP* mutant where a combination of these effectors are prevalent such as within a mammalian host.

### Increased susceptibility of *bpiP* mutant to macrophages/dendritic cells

**Fig 14:**
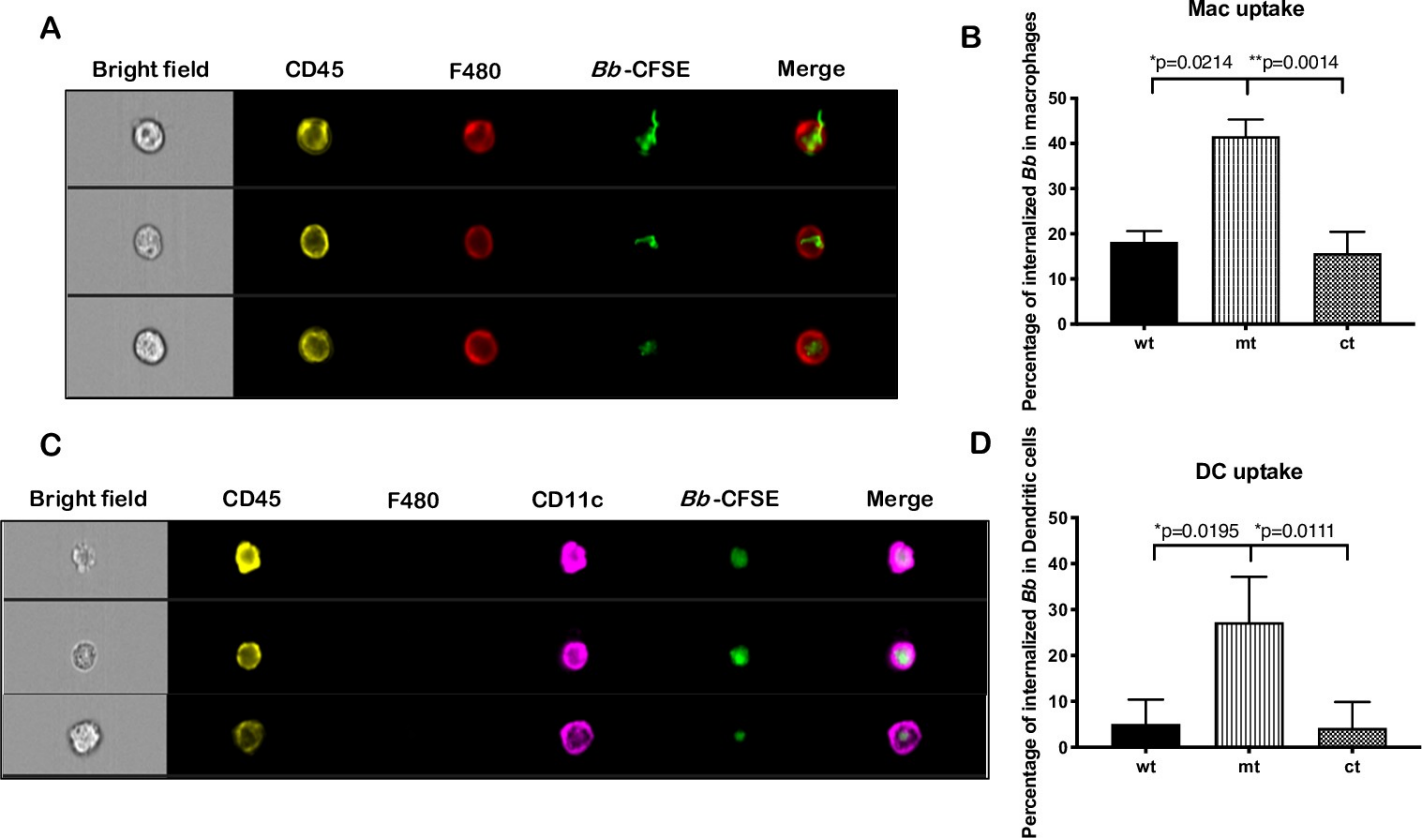
Phagocytosis of *bpiP* mt by activated macrophages and dendritic cells. All three strains (wt, mt and ct) were labeled with CFSE dye as described in the Materials and Methods sections and incubated with activated macrophages or dendritic cells for 10 mins. The infected cells were then harvested and stained for macrophage positive markers CD45 and F4/80 using antibodies conjugated to the fluorophores PE and APC respectively. Macrophages were identified as CD45^+^ (Ex_(max)/_Em_(max)_: 565/578; channel 3) /F480^+^ (Ex_(max)/_Em_(max)_: 652/660; channel 11) cells while DC were identified as CD45^+^/CD11c^+^ (conjugated to the flurophore BV421, Ex_(max)/_Em_(max)_: 495/520; channel 7) but F480 negative cells. The samples were analyzed by Image Flow Cytometry with **(A)** macrophage markers detected using channels 3 and 11 corresponding to excitation wavelengths of 488nm and 642 nm, respectively and **(C)** DC markers were detected using channel 7 with an excitation wavelength of 405nm. CFSE-labeled *Bb* was detected using channel 2 (*Bb*/CFSE Ex_(max)/_Em_(max)_: 495/520) with an excitation wavelength of 488nm. Data shown are means of three technical replicates with error bars representing Standard Error Mean. Pictures are representative of data obtained from one of three independent experiments. The percentage of internalized *Bb* within **(B)** macrophages and **(D)** dendritic cells was quantified and analyzed statistically using unpaired *t* test. The asterisks indicate levels of significance as follows: **, *p* <0.01; *, *p* <0.05.

There was a significant reduction in the number of mt spirochetes detected in C3H/HeN mice by qPCR at day 14 post-infection although there were viable spirochetes in select tissues isolated from infected mice (Fig 7A). This significant reduction in *bpiP* burden indicated the inhibitory role of cellular and soluble mediators of innate immunity that limited the colonization of the mt compared wt or ct strains. We, therefore, used Imaging Flow Cytometry that allows for both qualitative (Fig 14A and 14C) and quantitative measurement (Fig 14B and 14D) of levels of uptake of *Bb* by macrophage and dendritic cells [41]. Quantitative measurement of uptake of *Bb* revealed that the *bpiP* mt has higher percentage of phagocytosis by activated macrophages (F480^+^) and dendritic cells (CD11c^+^) than control strains, suggesting that the increased clearance of *bpiP* mutant during early stages of infection could partly explain the significant reduction in *bpiP* burden in infected C3H/HeN mice by quantitative real time PCR (Fig 7A) even at day 14 post-infection. These observations demonstrate that lack of *bpiP* results in increased uptake of mt spirochetes by cellular mediators of innate immunity during the initial stage of infection and likely contribute to reduce infectivity of *bpiP* mt in immunocompetent mice. Therefore, its survival through tick and mammalian phases of infection is reliant on appropriate levels of expression of multiple determinants conferring resistance to these host-derived factors.

### Complement dependent killing of *bpiP* mutant

**Fig 15:**
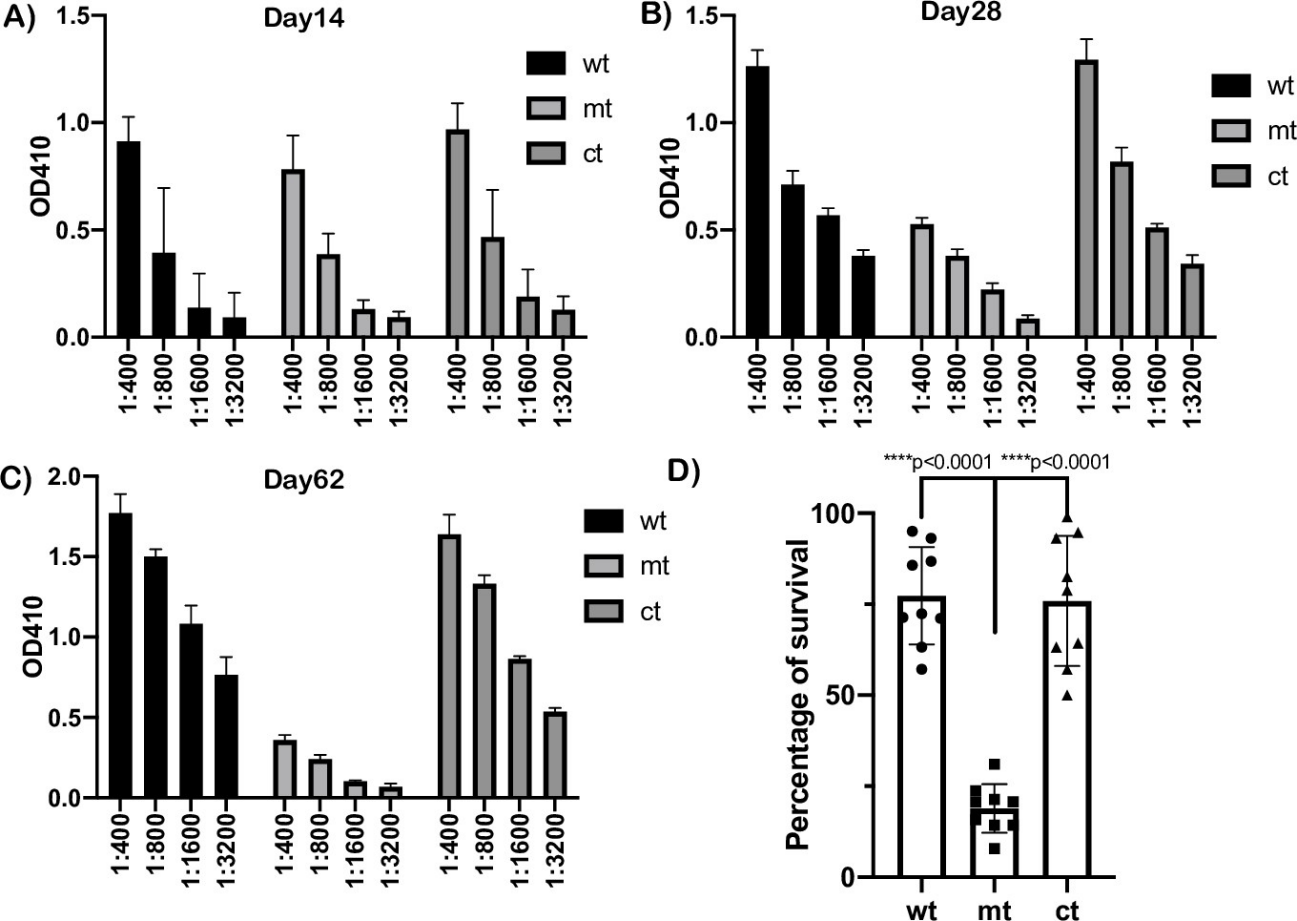
Seroconversion and borrelicidal antibody responses in C3H/HeN mice. Serological response in C3H/HeN mice infected with wt, mt and ct by needle inoculation at day **(A)** 14, **(B)** 28 and **(C)** 62 post-infection. Antibody responses in serum from wt, mt and ct infected mice were measured using ELISA plates with whole wt *Bb* lysate coated as antigen. Serial two-fold dilution starting at 1:400 dilution was used and plates developed using HRPO-conjugated anti-mouse whole Ig antibodies and plates developed using ABTS as substrate and absorbance measured at OD_410_ nm using BIOTEK Synergy ELISA reader. Data shown are means of three technical replicates with error bars representing Standard Error Mean and is representative of one of the three experiments. **(D)** Wt, mt and ct strains were treated with intact or heat inactivated guinea pig complement in the presence day 14 serum from C3H/HeN mice infected with the mt as described in the materials and Materials and Methods. Survival percentage of spirochetes was determined by number of motile spirochetes from intact complemented treated sample in comparison to heat inactivated sample. The levels of significance were determined using unpaired *t* test with GraphPad Prism software and a *p* value of less than 0.5 was considered as significant.

The development of the adaptive immune response as a contributing factor in reducing the survival of the *bpiP* mutant was predicted as a possible mechanism to reduce the *bpiP* burden quantitatively in immunocompetent mouse models of infection [51]. The level of antibody titers from C3H/HeN mice at day 14, 28 and 62 post-infection was determined by ELISA using whole cell lysates of wild type *Bb*. Consistent with above hypothesis, as shown in Fig 15A, the levels of antibody titers at day 14 was not statistically different in sera from mice infected with wt, mt or ct strains. However, the serum from mice infected with mt had significantly lower levels of antibodies at day 28 and 62 compared to the control strains consistent with reduced pathogen burden in these mice (Fig 15B and 15C). In order to determine if the levels of antibodies induced within 14 days were borrelicidal, we determined the complement-dependent killing of serum from mt infected mice. As shown in Fig 15D, the survival of mt was significantly lower compared to the control strains in the presence of guinea pig complement and day 14 serum from mice infected with mt. These observations indicate that the *bpiP* mt is unable to induce long lasting adaptive immune response and is cleared during the early stage of infection within the mammalian host due to complement mediated events.

### Attenuation of infectivity of *bpiP* mutant is not due to lack/loss of lp28-1/VlsE

**Fig 16:**
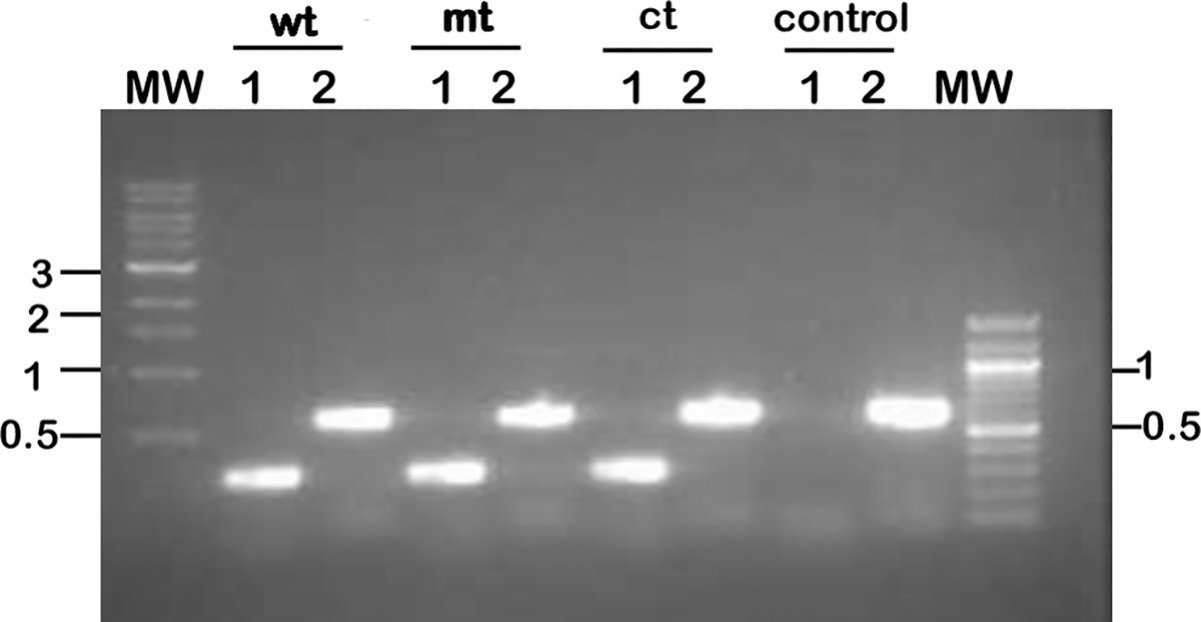
Verification of the presence of Lp28-1 and Lp25 in wt, mt, and ct strains. Total genomic DNA from wt, mt, ct and lp28-1- deficient clone of B31-A3 (negative control) was used to determine the presence of Lp28-1 (Lane 1) and Lp25 (Lane 2) by PCR using primers listed in the reference [52, 102]. Amplicons were separated using a 1% agarose gel and molecular size markers in kilobases are shown on both sides of the gel.

**Fig 17:**
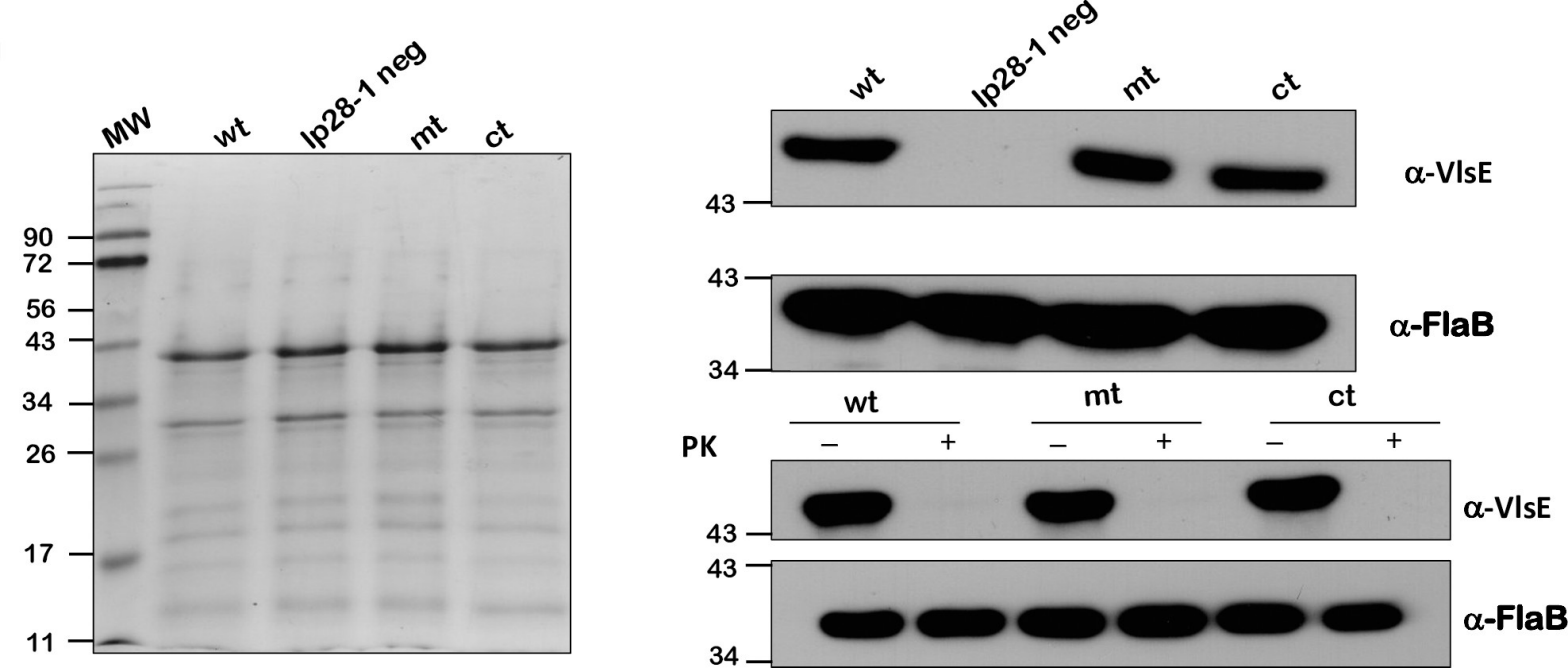
Levels of VlsE in wt, mt and ct strains. Coomassie blue stained SDS-PAGE gel of proteins from borrelial strains (wt (B31-A3), lp28-1 negative clone (B31-A3), mt and ct) grown under 32°C/pH7.6 in BSKII medium (**A**). Immunoblot analysis of lysates from the above borrelial strains with anti-VlsE serum (**B**) and following Proteinase K treatment of borrelial strains (**C**). Immunoblots using FlaB antibodies served as controls in both **B** and **C**. Molecular weight markers in kilodaltons are indicated to the left.

The *vls* locus encoded on the linear plasmid 28–1 has been shown to play a role in the persistence of *Bb* in reservoir hosts by contributing to antigenic variation and enabling the survival of *Bb* in the presence of an intact adaptive immune response [52, 53]. It is feasible that the loss of infectivity of *bpiP* mutant was due to lack of lp28-1 and surface expression of VlsE that may impact the ability of the *bpiP* mutant to colonize immunocompetent C3H/HeN mice. Both lp28-1 and lp25 were readily detected by PCR using plasmid specific primers while a clonal isolate of Bb-B31 strain lacking lp28-1 did not have an amplicon serving as a negative control (Fig 16). Immunoblot analysis of protein lysates from wt, mt and ct had similar levels of VlsE while the clonal isolate lacking lp28-1 did not show any reactivity (Fig 17B). Immunoblot analysis of spirochetes subjected to Proteinase K (PK) treatment revealed similar surface exposure of VlsE (Fig 17C). There were no changes in the levels of FlaB with or without PK treatment as FlaB is a periplasmic protein inaccessible to Proteinase K. These studies clearly demonstrate that the loss of infectivity of *bpiP* mutant in immunocompetent mice is not due the lack of lp28-1, lp25, levels and location of VlsE.

## Discussion

The Peptidoglycan (PG) cell wall is an essential component of the cell envelope contributing to the survival of bacterial pathogens under a variety of inhospitable environments [54]. Therefore, the biogenesis, composition and accessory determinants that provide structural and functional integrity to cell wall play a critical role during the colonization and persistence of *Bb* during the tick and mammalian phases of infection [19, 23]. Moreover, integration of synthesis and organization of multiple cell envelope/cell wall components with central and intermediate metabolism synchronized with regulatory effects on motility, morphology, and virulence underscore the importance of the role of these determinants in the pathophysiology of Lyme spirochetes. Recent findings that 1) discrete zones of PG are synthesized to elongate *Bb*; 2) PG is a persistent antigen in Lyme arthritis patients capable of inducing a pro-inflammatory response; and 3) PG fragments accumulate in the medium rather than recycled for generation of new PG have opened avenues to explore the role of pathogen-specific differences associated with cell wall or cell envelope of *Bb* as unique targets to interfere with Lyme disease pathogenesis [18, 25].

A major finding from this study is the role of a borrelial homolog, BpiP, annotated as OmpA-C like protein, in the survival of *Bb* in mammalian hosts and its role in contributing to the integrity of borrelial PG (Fig 1). Unlike other cannonical OmpA-C protein in *E*. *coli* that connects PG to the outer membrane, BpiP is localized to the protoplasmic cylinder suggesting the possibility of anchoring the PG to the inner membrane (Fig 2). In addition, BpiP-3X-FLAG-tag protein was detected with the periplast fraction although BpiP does not have a typical signal sequence to facilitate its secretion to the periplasmic space (Fig 2F). It should be pointed out the levels of BpiP were lower in the periplast fraction and it is feasible that BpiP is localized in the cytosol or in the inner membrane of *Bb* for interaction with L-Ornithine and may potentially serve to “trap and transport” this non-proteogenic amino acid following its biogenesis from citrulline due to the enzymatic functions of ornithine carbamoyltransferase (ArgF, BB0842). Alternatively, it is possible for BpiP to anchor the borrelial PG to the inner membrane or potentially sequester L-Ornithine assimilated from the host environment requiring BpiP localization within the periplasmic space. The signal-dependent synthesis (temperature, pH and stage of cell division) and partitioning of BpiP is unknown and additional studies are needed to delineate how BpiP influences various steps involved in the biogenesis of borrelial PG. The major flagellin of *Bb*, FlaB, also lacks a canonical signal sequence but is secreted to the periplasm via the Type III secretory pathway or by interaction with another periplasmic protein FlaA with a canonical signal sequence that facilitates its secretion into the periplasmic space [17, 55]. There are examples of non-flagellar proteins being secreted via the flagellar Type III secretory pathway [56]. It is possible that BpiP is chaperoned by another protein to the periplasmic space or utilizes known or unknown secretory pathways to be localized in the periplasmic space to interact with the pentapeptide of borrelial PG.

Consistent with prior structural analysis that predicted interaction of BpiP with L-Ornithine via a glutamic acid residue at position 315 [30], our cross-linking studies demonstrated that the recombinant BpiP with E315A or with 7 site-specific changes replacing the residues of the peptidoglycan-binding motif was unable to bind to borrelial PG unlike the native BpiP (Fig 3A and 3B). Moreover, BpiP interacted with borrelial PG but not with heterologous PG from *E*. *coli* and *B*. *subtilis* that had *meso*-DAP or L-Lysine as the third amino acid reflecting the specificity of BpiP-*Bb* PG interactions (Fig 3C and 3D). Therefore, BpiP appears to exert a pathogen-specific function consistent with the unique composition of the pentapeptide of *Bb* PG.

Biogenesis of PG in the absence of amino acid biosynthetic pathway/s for making L-Ornithine suggests the possibility that *Bb* acquires this non-proteogenic amino acid from the growth media or from host-derived sources or generates this molecule via an alternate pathway [2, 5]. Due to the non-proteogenic nature of L-Ornithine, it is unlikely that OppA-dependent peptide transport system is involved in the process of transporting L-Ornithine [12, 13]. Prior studies have shown no significant phenotypic differences in the phenotype of *Bb* following supplementation of L-Ornithine in borrelial growth medium [57]. The presence of a homolog, BB0842, annotated as ornithine carbamoyltransferase (ArgF) suggests possibility of Arginine being converted to Citrulline, which in turn can be converted to L-Ornithine by ArgF [57, 58]. Moreover, the presence of ArcA (BB0841, arginine deaminase) and ArgF provides for a source of L-Ornithine for PG biogenesis [28, 40, 57, 59–61]. Alternatively, it is possible that *Bb* is able to acquire host derived L- Ornithine—a naturally occurring non-proteogenic amino acid obtained via the actions of Arginase on Arginine that is prevalent in different hosts. Similarly, mammalian sources of N-acetyl hexosamine could be harnessed during the biogenesis of borrelial PG.

Sequence analysis of BpiP homologs present in the Relapsing Fever (RF) spirochetes (*B*. *hermsi* and *B*. *turicatae*) reveal the presence of an alanine at position 315 which raised the issue of levels of interactions between these homologs with L-Ornithine as the third amino acid in the pentapeptide of RF PG. In addition to this difference, RF BpiP homologs also have different amino acids within the PG binding domain that could contribute to the interaction between RF BpiP and its PG (Fig 1A). It is also possible that the RF spirochetes carry L-Lysine as the third amino acid and therefore A_315_ may have little impact on the interactions between the RF BpiP and its PG. Additional studies are warranted to explore the significance of the difference between BpiP homologs of LD and RF spirochetes.

In addition to the source of L-Ornithine, bioinformatic analysis of genomes of *B*. *burgdorferi* revealed presence of several conserved homologs encoding for enzymes that sequentially add various components of the PG [19, 23]. Among these homologs, borrelial MurE (BB0201) is annotated as UDP-N-acetylmuramyl tripeptide synthetase and as UDP-N-acetylmuramoylalanyl D-glutamate-2,6-diaminopimelate ligase while MurE homolog in *B*. *burgdorferi* strain CA382 is annotated as UDP-N-acetylmuramoylalanyl D-glutamate-L-ornithine ligase. While there are multiple annotations of MurE in different *Bb* strains, biochemical evidence show that L- Ornithine is incorporated as the third amino acid in the pentapeptide which is key to the interaction of BpiP with borrelial PG [2, 25].

The significance of the presence of L-Ornithine as the third amino acid has implications not only for the interactions of BpiP with the PG but also in the sensitivity to host- and pathogen-derived PG degrading enzymes generating muropeptides capable of stimulating Pathogen Recognition Receptors (PPRs) in the mammalian host. Previous studies have shown that borrelial muropeptides stimulate human Nod2 reporter cell line and a specific inhibitor of the Nod2 signaling pathway inhibited its activation as measured by NF-κB activation [25, 62]. It is feasible that PG-binding proteins, like BpiP, while presumably anchoring PG to the inner membrane could limit access to endopeptidases that destabilize the structural integrity of the cell wall. Glycosidases, amidases and peptidases are three major classes of pathogen-encoded proteins that cleave bonds within PG. Compared to 5 amidases–enzymes that cleave polymeric peptidoglycan–present in *E*. *coli* (Ami A, B, C, D and AmpD), Lyme spirochetes have been shown to encode for one amidase, N-acetylmuramyl-L-alanine amidase (MurNac LAA) capable of hydrolyzing the amide bond between N-acetylmuramic acid and the N-terminal L-alanine residue of the stem peptide [63]. Moreover, muropeptides accumulated in the medium due to the absence of homologs that contribute to recycling induced Nod2-dependent signaling leading to expression of select cytokines [25, 62]. Two putative endopeptidases classified as M23 peptidase domain-containing proteins with similarity to murein DD-endopeptidase (MepM) and murein hydrolase activator NlpD with a LysM domain are present in the genome of *Bb* B31-A3 strain. However, there are no reports on the role of these proteins in the cleavage of borrelial pentapeptide crosslinks [2, 64, 65]. Since the *in vitro* phenotype of *bpiP* mutant was similar to control strains, it is unlikely that pathogen-derived enzymes impacted *bpiP* mutant while host-derived PG degrading enzymes could play a role in *bpiP* survival in mice.

The cell wall biogenesis begins via mevalonate pathway with acetate being converted acetyl-phosphate and subsequently to acetyl coA by acetate kinase (AckA) and phosphate acetyl transferase (Pta), respectively, [19, 22, 23]. In addition to driving the mevalonate pathway, levels of acetate (and other Short Chain Fatty Acids (SCFAs) such as butyrate and propionate) induce metabolic changes that alter the levels of several lipoproteins on the surface of the outer membrane via a relatively undefined acid-stress response that drive gene expression by modulating levels of sigma factor, RpoS [23]. The metabolic overlap mediated by host-derived signals/nutrients (such as SCFAs) during initiation of cell wall biogenesis and levels of major surface exposed lipoproteins imply integration of multiple pathways for remodeling the cell envelope/cell wall consistent with the adaptive capabilities of *Bb* in different hosts [19–21]. Transcriptional levels of *bpiP* were elevated in fed nymphs and under *in vitro* conditions mimicking fed nymphs (Fig 4) suggesting the possibility of levels of BpiP coinciding with PG synthesis or cell division [36].

The mutant lacking *bpiP* had no significant differences in *in vitro* growth rates under conditions mimicking different stages of tick infection (S4 Fig) or in the levels of select borrelial proteins in the mutant (Fig 6). However, the burden of *bpiP* mutant was significantly lower in immunocompetant C3H/HeN mice at day 14, 28 or 62 post-infection (Fig 7) while viable spirochetes at day 28 and 62 post-infection were not readily detected in infected tissues suggesting a role for the adaptive immune response contributing to the susceptibility of mutant spirochetes (Table 1 and Fig 7B and 7C). While complement-dependent killing of mt spirochetes was also observed with day 14 mouse serum (Fig 15D), most of the tissues from C3H/HeN mice had viable spirochetes at day 14 post-infection although there was a significant reduction in number of mt spirochetes (Fig 7A). The ability of *bpiP* mt to survive in the immunodeficient SCID mice but not in the immunocompetent BALB/c mice pointed to adaptive immune response of the host in the susceptibility of *bpiP* mt (Table 2 and Fig 8A and 8B). The likelihood of loss of Lp28-1 encoding for VlsE was also ruled out as wt, mt and ct strains had Lp28-1 and expressed similar levels of VlsE on the surface (Figs 16 and 17). It is interesting to speculate that the lack of BpiP allows for the modulation of the host immune response to be amplified against unknown borrelial determinants that are differentially expressed between mt and the control strains impacting survival of *bpiP* mutant in immunocompetent mice.

There was a significant reduction in the mt burden in fed infected larvae and fed infected nymphs compared to the levels of wt and ct strains (Fig 9B and 9C). The ability of the naïve larvae to acquire mt spirochetes within 5 days post-infection from needle challenged mice was diminished and only two of 9 infected larvae out of the total 9 examined under the dark-field microscopy were positive for spirochetes and these observations were consistent with the quantitative real-time PCR analysis used to determine the mt burden. These findings reflect the reduced burden of *bpiP* mt in needle-infected C3H/HeN mice that limited the larvae from acquiring the mt at levels similar to wt or ct strains. These observations, however, were xenodiagnostic of infection in C3H/HeN mice where presence of *Bb* is detected by the feeding *I*. *scapularis* larvae (Fig 9B and 9E) [66, 67]. Moreover, there was a significant reduction in the mt burden in fed infected nymphs compared to those infected with wt or ct strains suggesting a lack of expansion of the mt spirochetes following ingestion of a blood meal by infected flat nymphs (Fig 9C). Although, one out of the three mice was positive for infection with the mt in select tissues, collectively the rates of transmission following challenge with infected nymphs was significantly lower for *bpiP* mt (Table 3 and Fig 9D). These studies demonstrate that absence of *bpiP* limits the survival of *Bb* in the mammalian hosts resulting in reduced acquisition/transmission of mt spirochetes via tick larvae and nymphs, respectively. The role of *bpiP* in the mammalian phase of infection can, therefore, be exploited to reduce the acquisition and transmission of *Bb* via ticks to naïve mammalian hosts.

We tested the effects of several stressors to determine the underlying basis for the inability of *bpiP* mutant to survive within mammalian hosts. Vancomycin was previously shown to be an antibiotic that reduces the cell wall stiffness/elasticity impacting shape and motility of *Bb* [46, 47]. Although the timing of BpiP synthesis and interaction with borrelial PG is unclear, *bpiP* mutant had increased sensitivity to vancomycin at 0.5 and 1μg/ml compared to the control strains (Fig 10A) and lower cfus/ml with 0.5 μg/ml of vancomycin (Fig 10B). Similarly, the *bpiP* mutant was sensitive to higher concentration of NaCl (200mM) in the BSK growth medium (Fig 11A) with reduced cfus/ml compared to the control strains (Fig 11B) and exhibit detectable morphological changes such as blebbing compared to the control strains. One possible explanation for this phenotype is the decreased integrity at the site of BpiP-PG interaction within the pentapeptide bridge altering the sensitivity to osmotic stress or morphology of spirochetes in the presence of increased levels of NaCl. Both these stressors did not completely inhibit the *bpiP* mutant suggesting that the changes in the cell wall stiffness/motility or ability to withstand osmotic stress alone is insufficient to contribute to the reduced infectivity of *bpiP* mutant.

We hypothesized that mediators of innate immune response due to macrophages, dendritic cells, among other cells, contribute to the significant decrease in the mt burden in C3H/HeN mice at day 14 post-infection before a robust adaptive immune response is established. Lysosomal extracts from bone marrow derived dendritic cells supplemented in borrelial growth medium reduced growth of *bpiP* mt significantly compared to the parental controls (Fig 12A and 12B). Moreover, antimicrobial peptide (AMP), LL37, the only member of the human cathelicidin family, significantly reduced the survival of *bpiP* mt compared to the control strains (Fig 13A and 13B). The mouse ortholog of human LL37, mCRAMP, had no significant effect on the *bpiP* mutant (S2 Fig) while it was effective against *E*. *coli*. These observations were similar to the lack of sensitivity of *p66* mutant in the presence of mCRAMP under *in vitro* growth conditions [68]. Since the positive charge of LL37 favors interaction with negatively charged phospholipids, it is feasible that the loss of *bpiP* impacts the borrelial cell envelope to facilitate the effects of LL37 on borrelial inner membrane [69]. Both macrophages and dendritic cells exhibited increased uptake of *bpiP* mutant suggesting that absence of BpiP results in changes that lead to increased uptake (Fig 14B and 14D). Mutants lacking key regulators of gene expression such as BadP or proteins critical for initial stages of colonization of mammalian host exhibit attenuation of infection due to enhanced phagocytosis and killing by macrophages and dendritic cells at the site of inoculation or infection [41]. However, the *bpiP* mutant had no growth deficit in SCID mice suggesting that soluble and cellular mediators of innate immunity is most likely insufficient to impact colonization while a significant growth deficit could be readily observed in immunocompetent mice as the adaptive immune response gets established. While the levels of *Bb*-specific antibody responses were similar against wt, mt and ct strains at day 14, it was lower at day 28 and 62 in mice infected with the mt consistent with the lack of survival of *bpiP* mutant (Fig 15A–15C). It is possible that the complement-dependent killing or the inability to withstand the effects of borrelicidal antibodies likely contribute to lack of survival of mt in immunocompetent mice (Fig 15D). It is also feasible that the lack of BpiP reduces the threshold of resistance to multiple antibacterial effectors targeting the PG cell wall and cell envelope of *Bb*. It has been shown patients with Lyme arthritis develop antibodies specific to borrelial PG [25]. It is likely that the absence of BpiP increases the borrelicidal effects of antibodies either alone or in conjunction with other mediators of innate immunity in mouse models of infection.

Host-derived enzymes that target the borrelial PG during the tick and mammalian phases of infection are being studied in greater detail [15, 64]. The immune deficiency (IMD) pathway and Toll pathway in the arthropods respond to diaminopimelic acid [70] and Lysine-type PG present in Gram-negative and Gram-positive bacteria, respectively [15, 71, 72]. Unlike DAP-type PG, it is unclear how the L-Ornithine type PG in *Bb* is sampled to activate the IMD pathway [15]. Similarly, there are 4 homologs of Peptidoglycan Recognition Proteins (PGRPs) in mice and humans with variable levels of expression, predicted domains and cellular locations that could contribute to the host response to borrelial PG [73–75]. Moreover, a lipoprotein, LipL21, bound and protected leptospiral PG from being digested as muropeptides prevented recognition of Leptospires via Nod1 or Nod2 signaling pathways although BpiP does not appear to be a lipoprotein [76].

**Fig 18:**
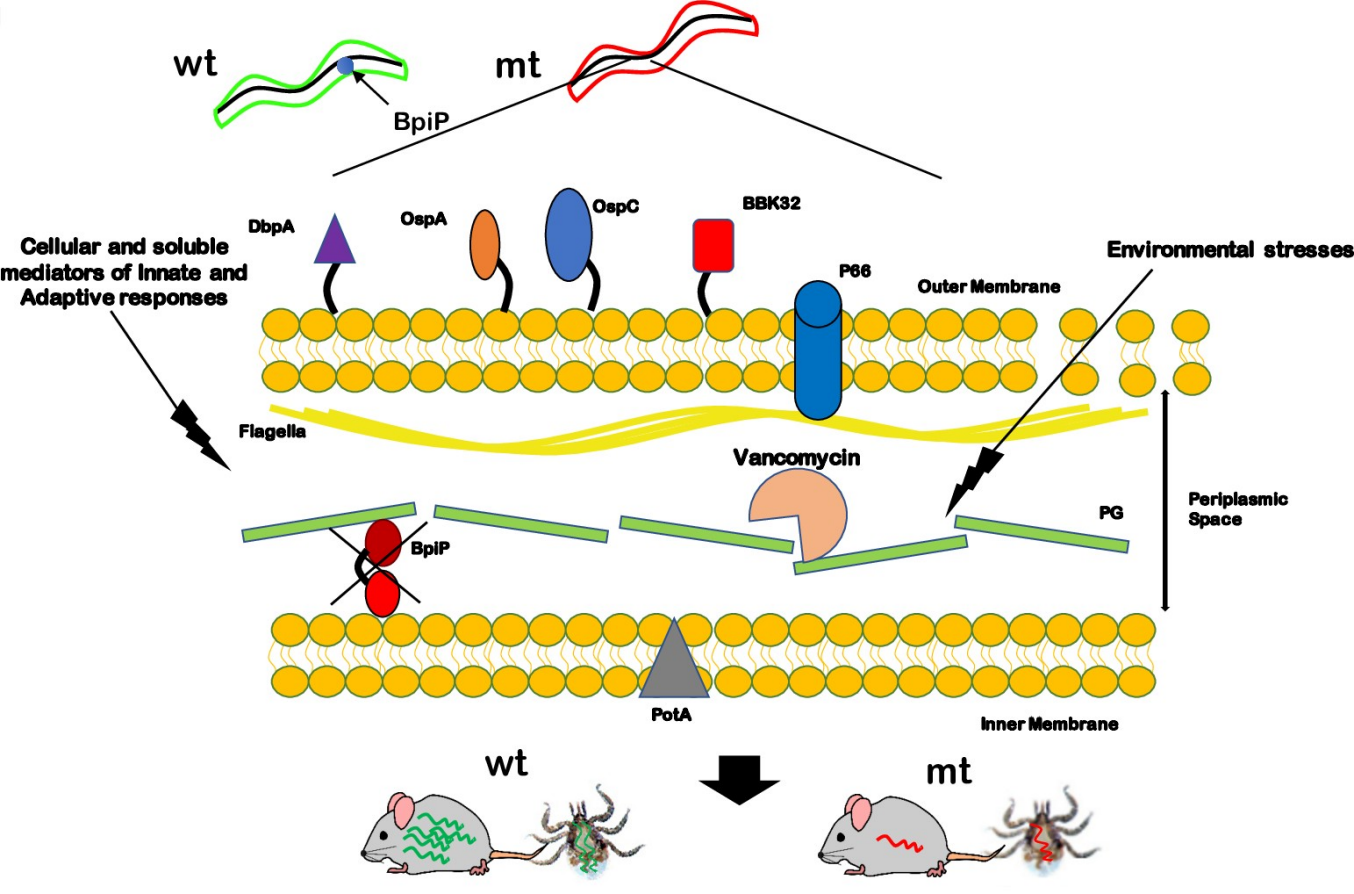
Graphical representation of role of BpiP. Structural, biochemical and genetic analysis reveal that BpiP interacts with the pentapeptide of borrelial PG stabilizing the cell wall integrity and most likely anchoring PG to the inner membrane. Absence of BpiP results in a mutant strain with a significant deficit in colonization of the mammalian host and the phenotypic analysis of *bpiP* mutant indicated increased sensitivity to a variety of environmental stressors as well as to soluble and cellular effectors of host immune response. The effects of pathogen-specific and host-derived PG degrading enzymes on borrelial PG are unknown and the role of BpiP conferring resistance to *Bb* against these enzymes is bound to open novel strategies to limit the survival of *Bb* during various stages of the infectious cycle.

In this study, we demonstrate the importance of BpiP in the contribution of cell wall/cell envelope in the survival of *Bb* within the mammalian hosts [77]. As summarized in Fig 16, the organization of the borrelial cell envelope and the unique composition of borrelial PG confer adaptive capabilities for *Bb* during the infectious cycle. Our studies show that BpiP plays a key role in the ability of *Bb* to survive in the mammalian host. Moreover, the nature of innate and adaptive immune response mounted against surface-exposed borrelial determinants and PG breakdown products impact the clearance or persistence of spirochetes in mammalian hosts (Fig 18). The molecular mechanisms that confer integrity to borrelial cell wall/cell envelope mediated via proteins like BpiP are likely to sustain the survival of Lyme spirochetes during difference phases of the infectious cycle. Strategies that subvert these mechanisms are likely to aid in developing novel preventive and therapeutic options for control of Lyme disease.

## Materials and methods

### Ethics statement

All animal experiments were conducted following NIH guidelines for housing and care of laboratory animals and in accordance with protocols approved by the Institutional Animal Care and Use Committee (protocol number MU071) of the University of Texas at San Antonio (UTSA). Based on these guidelines, general condition and behavior of the animals were monitored by trained staff. The animal facilities at UTSA are part of Laboratory Animal Resources Center (LARC), which is an AAALAC International Accredited Unit. During tick infection phase, all animals were monitored every 12 hours and tick-challenged animals were housed under Biosafety Level 2 facility with restricted entry.

### Bacterial strains and growth conditions

A low passage infectious clonal isolate of *Borrelia burgdorferi* B31-A3 was used as the parental strain in all the studies (Table 4) [78]. Wild type (wt), mutant (mt) and complemented (ct) strains were propagated in liquid Barbour-Stoenner-Kelly (BSK II) media supplemented with 6% heat inactivated rabbit serum (Pel-Freez Biologicals, Rogers, AR) with appropriate antibiotics (Sigma-Aldrich, St. Louis, MO) as previously described [12, 19, 20, 38–40]. All borrelial strains were first grown at pH7.6/32°C (laboratory growth conditions) and when the cultures reached a density of 1×10^7^ spirochetes/ml, the bacteria were seeded into fresh BSK II medium at pH 7.6/23°C to mimic the midgut of un-fed tick. Once the cultures at un-fed tick mimicking conditions reached a density of 1–2×10^7^ spirochetes/ml, the spirochetes are seeded into fresh BSK II medium at pH 6.8/37°C mimicking the midgut of fed ticks. Total RNA or protein lysates were extracted/prepared from these cultures when the density of *Bb* reached 5x10^7^ cells or 1x10^8^ *Bb* /ml for transcriptional and translational analyses, respectively. Harvested cells were washed three times with Hanks Balanced Salt Solution (HBSS, HyClone, ThermoFisher Scientific, Waltham, MAA) and processed for extraction of RNA or proteins.

**Table 4:** Plasmids and strains used in this study.

Plasmid	Description	Source or reference
pCR®2.1-TOPO	PCR cloning vector, Amp^R^ Kan^R^	Invitrogen
pMAL-2c	Expression vector with a maltose binding protein tag, Amp^R^	Novagen
pSV001	BB0167 cloned into pCR2.1 Amp^R^ Kan^R^	This study
pSV002	BB0167 cloned into pMAL-2c	This study
pSV003	Upstream region of BB0167 in pCR2.1 Amp^R^ Kan^R^	This study
pSV004	Downstream region of BB0379 in pCR2.1 Amp^R^ Kan^R^	This study
pSV005	Deletion construct for generating *bpiP* strain Amp^R^ Kan^R^ Strep^R^	This study
pSV006	Cis-complement construct for generating *bpiP* strain Amp^R^ Kan^R^ Gent^R^	This study
pYC200	BB0167 E315A cloned into pMAL-2c	This study
pYC201	BB0167 7SDMs cloned into pMAL-2c	This study
pML102	Vector containing P_flgB_-Strep^R^ or P_flgB_-Gent^R^ with flanking SalI sites	[103]
pTM61	Borrelial shuttle vector expressing gfp	[44]
pYC153	Vector containing 1kb up of *bpiP* and *bpiP-FLAG-tag*	This study
pMCSG7	Plasmid expressing the C-terminal region of BpiP	[30]
***B*. *burgdorferi strains***	**Description**	**Source or reference**
wt	*Bb*, B31-A3 isolate with all infection-associated plasmids	[78]
mt	*bpiP* mutant strain with Strep^R^	This study
ct	*bpiP cis-complement* strain with Gent^R^	This study
***E*. *coli* strains**	**Description**	**Source or reference**
Top10	Cloning strain	[84]
Rosetta TM	Host for inducible expression protein	[84]

All *E*. *coli* strains were propagated in Luira-Bertani broth at 37°C with shaking and supplemented with the appropriate antibiotics (Sigma-Aldrich, St. Louis, MO) as previously described (Table 4) [12, 19, 38–40, 42, 43, 79–82].

### Generation of recombinant proteins and antibodies

Total genomic DNA was isolated from a low passage clonal isolate of *Bb* by phenol chloroform extraction followed by ethanol precipitation as previously described [12, 19, 38–40, 80]. Recombinant proteins and monospecific sera were generated as previously described [12, 19, 38, 40, 79, 80, 82, 83]. Briefly, total genomic DNA obtained from *B*. *burgdorferi* B31-A3 was used as a template to amplify genes of interest by PCR using primers listed in Table 5. Amplified genes were cloned into the cloning vector pCR2.1 TOPO using the TOPO 2.1 cloning kit (Invitrogen) and transformed into electrocompetent TOP 10 *E*. *coli* (Invitrogen, Carlsbad, CA). Positive colonies containing the amplified gene in pCR2.1 were selected via blue/white screening using plates supplemented with IPTG/X-gal followed by restriction enzyme digestion (EcoRI) of the plasmids from positive clones to confirm the presence of the insert of the correct size (New England Biolabs). The resulting plasmids were then digested with BamHI and SalI and cloned into the inducible expression vector pMAL-p2X (New England Biolabs). Site-specific substitutions replacing (1) E315 or (2) with 7 site directed mutations (7SDMs) replacing amino acids of PG-interacting motif of BpiP with alanines or glycine were performed using specific primers as listed in Table 5. The cloned expression vector was transformed into the *E*. *coli* expression host, Rosetta (Novagen). Protein expression was induced with 1mM IPTG for 4 hours at 37°C in a 500ml flask and the culture was pelleted by centrifugation for 10 min at 3000 x *g* (SORVALL, F13S rotor) followed by resuspension in Low Salt Buffer (LSB, 10mM phosphate buffer, pH7.0 containing 30mM NaCl and 10mM β-mercaptoethanol). Resuspended cells were then lysed using a French press. The resulting lysate was then spun down at 4,000 x *g* for 30 minutes and supernatant was loaded on to a column with amylose beads (New England Biolabs) and purified using the NGC chromatography system [84]. The column was washed with buffer A (LSB) and proteins were eluted with buffer B (LSB containing 50mM maltose). Fractions were analyzed on a 12% SDS PAGE gel. Maltose binding protein (MBP) was cleaved from purified recombinant BpiP proteins (wild type, E315A or 7SDMs) in LSB by incubating with TEV protease tagged with 7X Histidine at 4°C, overnight in TEV protease buffer (50mM Tris-HCl, 0.5mM EDTA, 1mM DTT at pH 7.5). TEV protease was removed using NEBExpress Ni Spin column as per the manufacturer’s protocol and MBP was then removed by repeated passage over amylose beads (New England Biolabs). The top aqueous fractions with only the BpiP proteins were collected, concentrated using centrifugal filter devices (Millipore) and separated on 12% SDS-PAGE gel, transferred to PVDF membrane. Immunoblot analysis was performed to evaluate the efficacy of cleavage of the MBP using antibodies directed against either MBP or BpiP (S1 Fig).

**Table 5:** Primers list.

Name	Sequence 5 ‘to 3’
Flab_F_qPCR	CAGCTAATGTTGCAAATCTTTTCTCT
Flab_R_qPCR	TTCCTGTTGAACACCCTCTTGA
β-Actin_F_qPCR	CAAGTCATCACTATTGGCAACGA
β-Actin_R_qPCR	CCAACAAGGAAGGCTGGAAAA
BpiP_F_del_up	ACGCGGATCCCCAGCTATAAGCTGCACTATC
BpiP_R_del_up	ACGCGTCGACCATCTTAAGTGTCGACATGGT
BpiP_F_del_down	ACGCGTCGACACTACACCATGTCGACACTTA
BpiP_R_del_down	ACGCGCGGCCGCGTCTCTCTTTATAAAGTTC
BpiP_F_external	TCAGATCAGAACAACATATTAAATAGC
BpiP_R_external	AGATTGCTAGGGAGAGACTTTTAGCTA
BpiP_F_qPCR	GACATACTGAGCAATTTGGATTGG
BpiP_R_qPCR	ATTGCACGAGCTCTTTTTTCAGA
addA_F_qPCR	TAACGCCACGGAATGATGTC
addA_R_qPCR	TCTCCGCGCTGTAGAAGTCA
BpiP_F_BamHI	ACGCGGATCCTTGTTGTTATTATTTTTAATTC
BpiP_R_SalI	ACGCGTCGACGTTATTTAATATTGTAATTTCTAC
BpiP_F_NdeI	ACGCCATATGTTGTTGTTATTATTTTTAATTC
BpiP_R_NotI	ACGCGCGGCCGCGTTATTTAATATTGTAATTTCTAC
BpiP E315A F	CTAATAGAAGGACATACTGCGCAATTTGGATTGGAAGAA
BpiP E315A R	TTCTTCCAATCCAAATTGCGCAGTATGTCCTTCTATTAG
BpiP 7SDMs F	CACGAGGCTGCTGAAAAAGCTGGACGTGCTGCTGGAAATTATGCTA TAAAAATGAAAGTA
BpiP 7SDMs R	TACTTTCATTTTTATAGCATAATTTCCAGCAGCACGTCCAGCTTTTTC AGCAGCCTCGTG
BpiP NheI F	ACGCCATAGTGCTAGCTTGTTGTTATTATTTTTAATTC
BpiP XhoI R	ACGCGCGGCCGCGGTACCTTAGTGGTGGTGGTGGTGGTGGTGGTGGTGGTGT TATTTAATATTGTAATTTCTAC
Lp25-F	ATG GGT AAA ATA TTA TTT TTT GG
Lp25-R	AAG ATT GTA TTT TGG CAA AAA ATT TTC
Lp28-1F	ATG AAC AAA AAA TTT TCT ATT TC
Lp28-1R	GTT GCT TTT GCA ATA TGA ATA GG

Recombinant C-terminal region A260-N380 of BpiP cloned into plasmid pMCSG7 with an N-terminal His-tag (BbOmpA^CTD^) was expressed and purified using Ni-NTA column [30] and the His-tag cleaved with TEV proteas. Recombinant C-terminal region of BpiP without His-tag was then emulsified in equal volume of Titermax gold adjuvant, respectively and used to immunize six to eight weeks old BALB/c mice (*n* = 3) with 50 micrograms/mouse (day 0). Boosters were administered at day 14, and 21 and serum was collected on day 28.

### Isolation of Peptidoglycan (PG) from *B*. *burgdorferi*

Wild type *Bb* (B31-A3) was grown in BSKII medium (1L) supplemented with 6% normal rabbit serum at pH 7.6/32°C to a density of 1x10^8^ *Bb*/ml and harvested by centrifugation. The pellet was resuspended in 20 ml ice-cold ultrapure water and was added to an equal volume of 8% SDS solution stirred in a beaker placed within a boiling water bath with continuous stirring for 1 hour [85–88]. After cooling to room temperature, PG was pelleted by ultracentrifugation at 130,000 x *g* using SORVALL Discovery 90 with Surespin 630 rotor at room temperature for 1 hour. The PG pellet was then resuspended in 20 ml of ultrapure water with vigorous vortexing and added to boiling 8% SDS solution and incubated for 15 min followed by ultracentrifugation at 130,000 × g at room temperature for 1 hour. After washing the PG with ultrapure water four times and pelleting by ultracentrifugation, PG was treated with 0.1 mg/ml α-amylase at 37°C for 2 hours followed by treatment with 0.2mg/ml pronase for 90 minutes at 60°C to hydrolyze proteins associated with PG. The mixture was added to an equal volume of boiling 8% SDS solution and incubated for 15 min in boiling water bath. The final pellet was then washed four times with water as described above and pelleted at 130,000 × g at room temperature for 1 hour [89]. The final PG pellet was stored at 4°C for further analysis.

### Muramic Acid Assay for Determining PG concentration

Purified peptidoglycan (40 μl) was resuspended in ultrapure water (Sigma) and an equal volume of 5M H_2_SO_4_ solution was added and incubated at 90°C for 2 hours to hydrolyze the PG as described previously [89]. Muramic acid standard solutions (0–1 mM) were treated similarly to generate a standard curve. After incubation, 180μl of ultrapure water and 90μl of 10M NaOH solution were added and incubated at 37°C for 30 mins to release lactic acid from muramic acid residues of PG. Then transfer 80 μl of hydrolyzed samples to clean glass tubes in triplicates and add 500 μl concentrated H_2_SO_4_ (18.8 M). Incubate glass tubes in a boiling-water bath for 5 mins and after tubes cooled down to room temperature, five μl of 4% CuSO4 solution and 10 μl 1.5% 4-phenylphenol solution were added and samples were incubated at 30°C for 30 mins and absorbance measured at 570nm. Peptidoglycan muramic acid units were determined using standard curve.

### Crosslinking of various PG with BpiP

Recombinant BpiP (100 μg/ml) was incubated with PG from *Bb*, *E*. *coli* (InvivoGen) or *B*. *subtilis* (Sigma) (200 μM muramic acid units of peptidoglycan) in Buffer A (40mM sodium phosphate [pH 6.5] with 0.5% Tween 20) on ice for 2 hrs [85, 86, 89]. An equal volume of Buffer B (50mM sodium phosphate [pH8.5] containing DTSSP (10mM) on ice for 30 mins was added followed by centrifugation at 21, 000 x *g* to pull down the cross-linked complexes. The samples were mixed with 2X SDS-PAGE sample buffer with 10% β-mercaptoethanol (β-ME) and boiled at 95°C for 5 minutes and the supernatant were analyzed on SDS-12% PAGE gel and transferred to PVDF membranes. The membranes were developed with mouse antiserum directed at C-terminal region of BpiP, goat anti-mouse IgG conjugated to HRPO and developed using ECL. BpiP-PG crosslinked complexes are unable to enter the gel but the cleavage of the cross-linker by β-mercaptoethanol treatment prior to electrophoresis facilitates analysis of the interactions of wild type BpiP or BpiP with site-specific changes with borrelial or heterologous PG in the supernatant as described in the flow chart in S3 Fig [86, 87, 89].

### Partitioning *Bb* cell envelope

The isolation of *Bb* outer membrane vesicle (OMV) and protoplasmic cylinder (PC) were isolated using a modified method as described previously [90]. Briefly, 1x10^9^ *Bb* cell were harvested by centrifugation and washed twice by 1x PBS (pH 7.4) and the pellet was re-suspended in 30 ml of ice-cold 25mM citrate buffer (pH 3.2). The mixture was incubated at room temperature for 2 hours and vortexed for 1 min every 30 mins. After incubation, the cells were pelleted at 20,000 x *g* for 30 mins and re-suspended in 3 ml of 25mM citrate buffer and layered onto a discontinuous sucrose gradient which composed of 56%, 42% and 25% sucrose. Then the whole gradient was centrifuged at 100,000 x *g* for 16 hours at 4°C using Beckman SW28 swing bucket. After centrifugation, the OMV fraction (upper band) and the PC fraction (lower band) were removed and the final fractions were stored in SDS loading buffer and used for further immunoblot analysis. Periplasmic and spheroplasmic fractions of *Bb* expressing pYC153 as reported previously [91, 92]. Briefly, 2x10^8^ *Bb* were treated with periplasting buffer (200mM Tris-HCl pH7.5; 50mM KCl, 1mM EDTA and 30units/μl of Ready-Lyse Lysosyme (Lucigen) and Omnicleave endonuclease. The cells were treated with sterile water and centrifuged to obtain the periplasmic fraction and the pellet treated with Peripreps Lysis buffer (10mNM Tris-HCl (pH7.5), 50mM KCl, 1mM EDTA and 0.1% dexoycholate, 10mM MgCl_2_). The supernatants were collected as spheroplasts. All samples were analyzed by immunoblot analysis using mouse anti-FLAG-tag, rabbit anti-Green Fluorescent Protein (gfp) and anti-FlaB monoclonal antibodies.

### Generation of *bpiP* mutant, cis- and trans-complemented strains

The low passage *Bb* B31-A3 strain described above was used for all genetic manipulations. Deletion of *bpiP* was performed as previously described [38–40, 79]. A suicide vector that contained homologous regions spanning 1,000 base pairs up- and down-stream of *bpiP* was used to replace the native copy of *bpiP* gene by allelic exchange with selectable streptomycin resistance marker under the control of the constitutive borrelial promoter P_*flgB*_. The up- and down-stream regions were amplified using primers listed in Table 5 and cloned into pCR2.1 cloning vector. The two fragments were then cloned together using the engineered restriction enzyme sites on the primers and those present in the pCR2.1 vector. An engineered Sal1 site between the two fragments was used to clone the streptomycin resistance marker as described above. The plasmid DNA was linearized with Sca1 to facilitate homologous recombination via double cross over events following electrotransformation of *Bb* B31-A3 strain as described previously [43, 78, 89, 93, 94]. Mutant colonies of *Bb* were obtained by plating in BSKII agar overlays in the presence of appropriate concentrations of antibiotics and colonies were screened by PCR and plasmid profile determined to be identical to parental strain.

Complementation of *bpiP* was performed using the same methods described above except the primers were designed for both upstream region and the *bpiP* gene. To select for proper complementation, a gentamicin resistance marker, *aacC1* under the control of the P_*flgB*_ promotor, was cloned downstream of the *bpiP* gene.

The trans-complemented strain was generated by transforming the *bpiP* mutant using a borrelial shuttle vector pYC153 obtained by cloning 1kb upstream of *bpiP* and *bpiP-*3XFLAG-tag at the Kpn1 site present in the plasmid pTM61[44].

Colonies were isolated and screened in the same manner as described above for *bpiP* mutants. All *Bb* strains generated as part of this study subjected to whole genome sequencing at the UTSA genome sequencing core and were found to have identical plasmid profile. Analysis of the sequence of the borrelial chromosome confirmed the location of the deletion/cis-complementation consistent with the region within plasmid constructs used to generate these strains.

### Sodium dodecyl sulfate (SDS)-polyacrylamide gel electrophoresis (PAGE) and Immunoblot analysis

Total borrelial lysates were obtained by harvesting the spirochetes as described above followed by separation on an 12% SDS PAGE gel. To ensure equal loading the gel was either stained with Coomassie Brilliant Blue (ThermoFisher) or transferred to PVDF membrane and probed with antibodies specific for borrelial FlaB as previously described [45, 82]. Proteinase K treatment of spirochetes was done as reported previously [95] Immunoblot analysis was performed as previously described with monospecific antisera from mice, rats, or rabbits generated against various borrelial proteins [41]. Blots were developed using appropriate HRP-conjugated secondary antibody (GE Healthcare) and ECL substrate (Thermo Fisher) followed by exposure to X-ray film.

### *in vitro* growth phenotype of borrelial mutants

Wt, mt and ct strains were diluted from cultures at pH7.6/32°C and re-seeded at 5×10^5^ spirochete/ml in fresh BSK II medium under growth conditions reflecting laboratory (pH 7.6/32°C), tick mid-gut before (pH 7.6/23°C) and after (pH 6.8/37°C) a blood meal. Cultures were enumerated every 24 hours using dark field microscopy using motility as a measure of viability. The cultures were grown in triplicate, with three independent biological samples. Error bars indicate standard error. Levels of significance were determined using unpaired *t* test with GraphPad Prism software (Graph-Pad Software, Inc., San Diego, CA) and a *p* value of less than 0.05 was considered significant.

### Complement-dependent killing of *Bb*

The anti-borrelial serum used in this study was from mice infected with the mt strain (*n* = 9; 14 days post-infection), and the borrelicidal activity was determined as described previously [96–98]. Briefly, 50 μL of diluted mouse serum (1:80) was mixed with 10 μL of guinea pig complement (guinea pig complement, Sigma-Aldrich, # S1639) or heat-inactivated guinea pig complement (negative control) with wt, mt or ct strains (5×10^6^ spirochete/ml) in 40 μL of BSK II (pH 7.6) medium and then incubated at 32°C for 24 h. Motile spirochetes following treatment were enumerated using dark-field microscopy. The survival percentage was calculated using the numbers of motile spirochetes from serum-treated samples to those with heat-inactivated complement (negative control). Error bars indicate standard error. Levels of significance were determined using unpaired *t* test with GraphPad Prism software and a *p* value of less than 0.05 was considered significant.

### Sensitivity assays

#### Antibiotics

Wt, mt and ct strains were diluted from stationary phase (1×10^8^ spirochete/ml) cultures (pH 7.6/32°C) and then re-seeded at 1×10^6^ spirochete/ml in fresh BSK II medium supplemented with vancomycin at pH 7.6/32°C in 96 well plate. Motile spirochetes were enumerated after 10 days of incubation. Colony forming units of cultures with 0 or 0.5 μg/ml of vancomycin at day 5 post-treatment were determined by diluting cultures of all three strains. The liquid cultures of all three strains were diluted 1:100,000 in 1X BSKII medium and plated as agar overlays with BSKII media with 0.84% agarose and the final CFU/ml were calculated after 10 days of incubation at 32°C with 1% CO_2_.

#### Osmolarity/ salt concentration

Wt, mt and ct strains were diluted from stationary phase (1×10^8^ spirochete/ml) cultures (pH 7.6/32°C) and then re-seeded at 1×10^6^ spirochete/ml in fresh BSK II medium supplemented with different concentrations of NaCl at pH 7.6/32°C. Cultures were enumerated every 24 hours using dark field microscopy. The liquid cultures of all three strains were diluted 1:100,000 in 1X BSKII medium and plated as agar overlays in BSKII media with 0.84% agarose and the final CFU/ml were calculated after 10 days of incubation at 32°C with 1% CO_2_. The cultures were grown in triplicate, with three independent trials. Error bars indicate standard error. Levels of significance were determined using unpaired *t* test with GraphPad Prism software and a *p* value of less than 0.05 was considered significant.

#### Mouse and tick infections

Unless stated otherwise, mice were infected by needle inoculation via intradermal route as previously described [40]. Six to eight weeks old C3H/HeN, BALC/c or SCID mice were injected intradermally with 10^5^ spirochetes per mouse. At 14, 28 or 62 days, post-infection, mice were euthanized and a piece of the abdominal skin, spleen, bladder, heart, left inguinal lymph node, and left tibiotarsal joint was crushed and cultivated in BSK-II media to isolate *Bb*. Cultures were scored for the presence of spirochetes 2 to 3 weeks later using dark field microscopy after one blind passage at day 5 post-isolation. Another piece of abdominal skin, half of the spleen, right inguinal lymph node and right tibiotarsal joint were used to isolate total DNA using High pure PCR template preparation kit (Roche) for qPCR analysis to enumerate the number of spirochetes in each tissue. Infectivity analysis using BALB/c and SCID mice were done at day 28 post-infection to determine the effects of adaptive immune response on the colonization capabilities of the *bpiP* mt.

*Ixodes scapularis* larvae were purchased from the tick rearing facility at Oklahoma State University (Stillwater, OK) and incubated in a chamber at 22°C with 99% humidity and a 15-hour light / 9-hour dark cycle. Larvae were allowed to feed on infected C3H/HeN mice with wt, mt or ct strains. Nine fed larvae were crushed to assess spirochete burden. The others were allowed to molt into nymphs and nymphs were then allowed to feed on naïve C3H/HeN mice to repletion (3–5 days). Fed nymphs recovered after feeding were washed with 3% H_2_O_2_, 70% ethanol and ddH_2_O sequentially and crushed with a small volume of BSKII medium and inoculated into 3ml of fresh BSK-II medium or used for extracting total genomic DNA. The media containing crushed ticks was then scored 1 to 2 weeks later for the presence of spirochetes by dark field microscopy. Recovered spirochetes were subjected to PCR analysis for confirmation of correct strains recovered from ticks. Total genomic DNA was extracted from fed ticks followed by quantitative real time PCR using primers specific for *Bb flaB*. The *flab* copies per tick was graphed and the difference between *Bb* colonization was determined using unpaired *t* test with GraphPad Prism software. A *p* value of less than 0.05 was considered significant.

### Killing assay with crude lysosomal extracts

C3H/HeN or C57BL/6 mice were used in these experiments and housed under pathogen-free conditions under institutionally recommended guidelines of Laboratory Animal Resource Center at The University of Texas at San Antonio. Murine bone marrow-derived dendritic cells (BMDC) cultures were established as described previously [99]. Briefly, bone marrow cells were flushed from the femurs and tibiae of C57BL/6 mice. BMDC were cultured in RPMI 1640 (Gibco Life Technologies) supplemented with 10% heat-inactivated fetal bovine serum (iFBS), 2 mM L-glutamine, 100U/ml penicillin, 100μg/ml of streptomycin, and 50 mM β-mercaptoethanol (complete medium). Cells were washed, counted, and plated in complete medium supplemented with 20ng/ml GM-CSF (PeproTech). One half of the medium was replaced every three days, and the cells were harvested on day 8. The cells were then purified by negative selection of F4/80^+^ cells using F4/80 biotin and anti-biotin beads (Miltenyi Biotec, Auburn, CA). The flow-through cells were further positively selected using magnetically labeled CD11c antibodies (Miltenyi Biotec).

Lysosomal extracts were prepared as described previously [99]. Lysosomal extraction buffer (1X Sigma-Aldrich) was added at 2ml per 3×10^8^ cells. DCs were homogenized with a PowerGen 700 homogenizer (Fisher scientific, Pittsbrugh, PA) using a 7-by110-mm homogenizer tip (Fisher Scientific). The homogenizer was passed through the cells 20 to 25 times. Cells were centrifuged at 1000 x *g* 10 min to remove intact cells and cellular debris. Then the supernatants were collected and centrifuged for 20 min at 20,000 x *g* to pellet lysosomes. The pellet containing lysosomes was resuspended in 1 mL of extraction buffer. The sample was then sonicated for 20 s at a setting of 40% on a model 500 Sonicator (Fisher scientific) to obtain the final crude lysosomal extract.

Wt, mt and ct spirochetes grown in BSKII media were harvested (1x10^6^ *Bb*) and resuspended in 1mL of new BSKII media (pH 6.8) supplemented with or without 10% lysosomal extract and incubated at 37°C, 1% CO_2_ for five days. The number of viable spirochetes was determined every 24 hours using dark field microscopy. The liquid cultures of all three strains were diluted 1:100,000 in 1X BSKII medium and plated as agar overlays in BSKII media with 0.84% agarose and the final CFU/ml were calculated after 10 days of incubation at 32°C with 1% CO_2_. Levels of significance were determined using unpaired *t* test with GraphPad Prism software and a *p* value of less than 0.05 was considered significant.

### Imaging flow cytometry to measure uptake of *Bb* by macrophage and dendritic cells

Uptake of wt, mt and ct strains of *bpiP* by macrophages and dendritic cells were performed using the ImageStream®X Mark II Imaging flow cytometer. Activated macrophages were obtained from six to eight-week old C3H/HeN mice after intraperitoneal injection of 1 ml of sterile 3% proteose peptone as previously described [79]. The mice were euthanized by CO_2_ asphyxiation after 4 days and 20 ml of DMEM (GIBCO) was injected to flush the cells from the peritoneal cavity. The cells were spun down and treated with 0.14M ammonium chloride to remove red blood cells followed by resuspension in DMEM supplemented with 10% iFBS. Cells were enumerated and then incubated with CFSE labeled *Bb* strains at a ratio of 1:1 in 50% cell culture medium (DMEM +10%iFBS) and 50% borrelial growth medium (BSK-II with 6% inactivated normal rabbit serum) at 37°C in 5% CO_2._ Cells were then spun down and resuspended in 2% paraformaldehyde (PFA) and sequentially washed two times with FACS buffer (PBS with 5% FBS). After washing, cells were resuspended in FC block at a 1:250 dilution (ThermoFisher) for 15 minutes. After incubation, the cells were washed twice with FACS buffer, resuspended in 50μl of FACS buffer and labeled with 0.8 μl of anti-CD45 PE, 0.4 μl of anti-F4/80 APC, 1.5 μl of anti-CD11C BV421 and 1 μl CD11b PE-Cy7 (ThermoFisher) and incubated at 4°C in the dark for 30 minutes. After incubation, the cells were washed twice with FACS buffer, resuspended in 2% PFA and samples were evaluated using the ImageStream®X Mark II Imaging flow cytometer at the UTSA Immune Defense core [45].

In order to enumerate the number of macrophages in the total cell population that were positive for *Bb* uptake, the total single cell population was first gated for CD45^+^ F4/80^+^ cells. This subset population was then gated for the percentage of cells that were also CFSE positive indicating cell with *Bb*. In order to determine the number of dendritic cells in the total cell population that were positive for *Bb* uptake, the total single cell population was first gated for CD45^+^ F4/80^-^ cell, then the subset population was then gated for CD11C^+^ cells. The remaining population was gated for the percentage of cells that were also CFSE positive due to *Bb* infection. Internalization of the spirochetes in macrophages and dendritic cells were determined using the Aminis IDEAS software suite.

### DNA sequencing for NGS

Sequencing of genomes was performed as previously described [41, 100]. Briefly, total genomic DNA from each strain was obtained through phenol chloroform extraction and checked for purity spectrophotometrically using NanoDrop microvolume spectrophotometer (ThermoFisher). Total gDNA was then submitted to the UTSA genome sequencing core for library preparation and sequencing on an Illumnia MiSeq. Resulting files were then uploaded to basespace and contigs were assembled using the velvet assembly program [101]. Contigs were then mapped to the reference chromosome and plasmids from NCBI using Geneious [2].

## Supporting information

S1 Fig Purification of recombinant BpiP proteins. Recombinant BpiP proteins (wild-type and with site-specific changes) fused to maltose binding protein (MBP) with an intervening TEV protease site were overexpressed and purified using amylose column in conjunction with FPLC (BIORAD). MBP was cleaved from BpiP using recombinant TEV protease fused to 7X-His-Tag bound to NiNTA beads. Recombiant BpiP was then concentrated from the flow-through fractions and was separated on 12% SDS-PAGE gel, transferred to PVDF membranes. Immunoblot analysis was performed using anti-Maltose serum or anti-BpiP serum generated against C-terminal region of BpiP followed by goat-anti-mouse IgG conjugated with HRPO and blots developed using Enhanced Chemiluminescence. Lane 1) BpiP-MBP, 2) BpiP-MBP + TEV treatment, 3) BpiP only, 4) E315A BpiP-MBP, 5) E315A BpiP-MBP +TEV treatment, 6) E315A BpiP only, 7) 7SDMs BpiP-MBP, 8) 7SDMs BpiP-MBP + TEV treatment, 9) 7SDMs BpiP only.
(TIF)

S2 Fig Sensitivity of *bpiP* mutant to murine antimicrobial peptides. All three strains (wt, mt and ct) were propagated at 10^5^/ml in BSKII growth medium at pH 6.8/32°C with 100 μg/ml of mCRAMP. Cells were enumerated every 24 hours using dark field microscopy. The cultures were grown in triplicate and one out of two independent experiments is shown. Statistical analysis of the difference in the number of wt and mt or ct and mt spirochetes was done by unpaired *t* test.
(TIF)

S3 Fig Flow-chart depicting the steps used to determine the interactions of BpiP with borrelial PG. BpiP (wild type and site-specifically altered proteins) were crosslinked to purified PG (devoid of any bound proteins) using the crosslinker DTSSP. The complexes were pulled down by high speed centrifugation and the samples boiled to release the bound proteins and supernatant was separated from PG and analyzed for levels of BpiP by immunoblot analysis using anti-BpiP serum generated against C-terminal region of BpiP.
(TIF)

S4 Fig Absence of an *in vitro* growth defect in *bpiP* mutant. Wild type (B31/A3), mt and ct strains were diluted from stationary phase (1×10^8^ bacteria ml^−1^) cultures, re-seeded at 5×10^5^ bacteria ml^−1^ in BSKII medium and enumerated every 24 hours using dark field microscopy under different growth conditions. The cultures were grown in triplicate, with three independent trials. Error bars indicate standard error. Levels of significance were determined using two-way ANOVA with α = 95%.
(TIF)
