# Supplementary figures and images for: Borrelia peptidoglycan interacting Protein (BpiP) contributes to the fitness of Borrelia burgdorferi against host-derived factors and influences virulence in mouse models of Lyme disease
Source: PLoS Pathog. 2021 Apr 21;17(4):e1009535. doi: 10.1371/journal.ppat.1009535 (PMC8092773; doi:10.1371/journal.ppat.1009535)

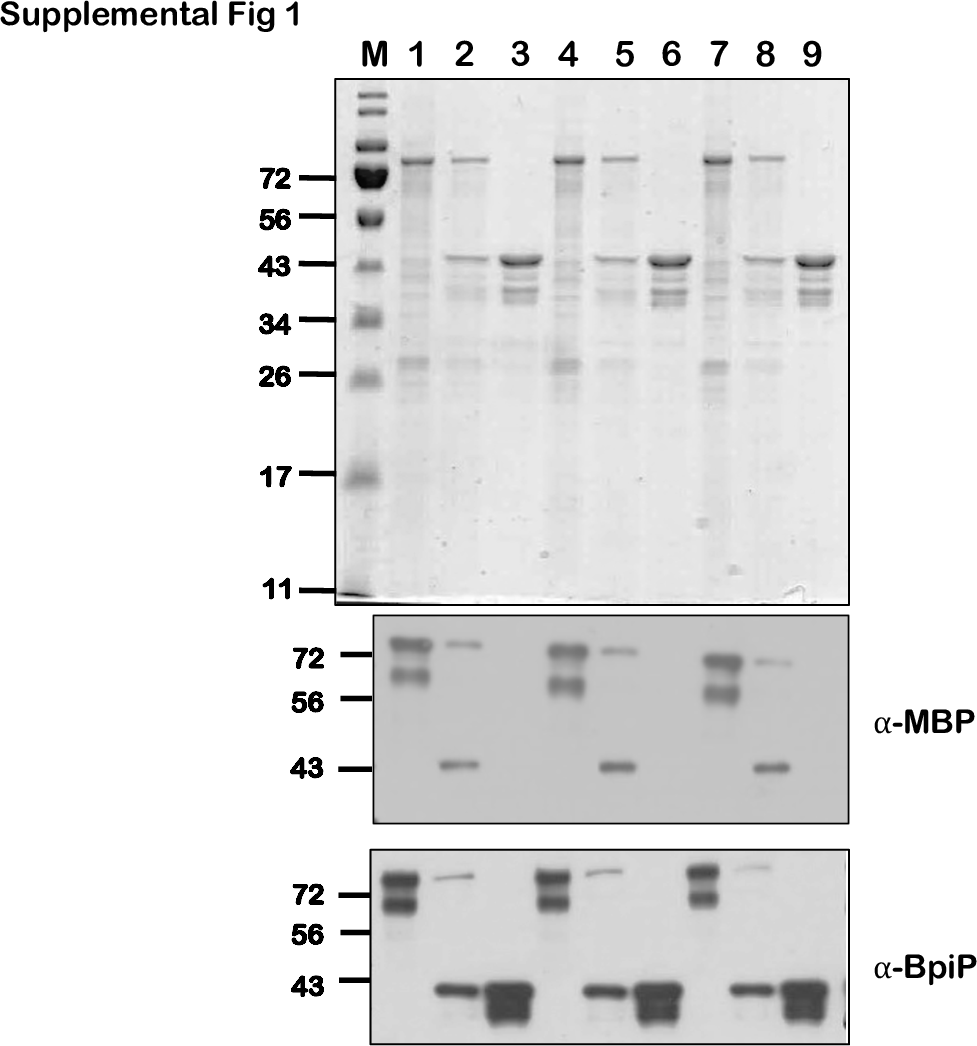

Supplement: S1 Fig — Recombinant BpiP proteins (wild-type and with site-specific changes) fused to maltose binding protein (MBP) with an intervening TEV protease site were overexpressed and purified using amylose column in conjunction with FPLC (BIORAD). MBP was cleaved from BpiP using recombinant TEV protease fused to 7X-His-Tag bound to NiNTA beads. Recombiant BpiP was then concentrated from the flow-through fractions and was separated on 12% SDS-PAGE gel, transferred to PVDF membranes. Immunoblot analysis was performed using anti-Maltose serum or anti-BpiP serum generated against C-terminal region of BpiP followed by goat-anti-mouse IgG conjugated with HRPO and blots developed using Enhanced Chemiluminescence. Lane 1) BpiP-MBP, 2) BpiP-MBP + TEV treatment, 3) BpiP only, 4) E315A BpiP-MBP, 5) E315A BpiP-MBP +TEV treatment, 6) E315A BpiP only, 7) 7SDMs BpiP-MBP, 8) 7SDMs BpiP-MBP + TEV treatment, 9) 7SDMs BpiP only. (TIF) [file ppat.1009535.s001.tif]

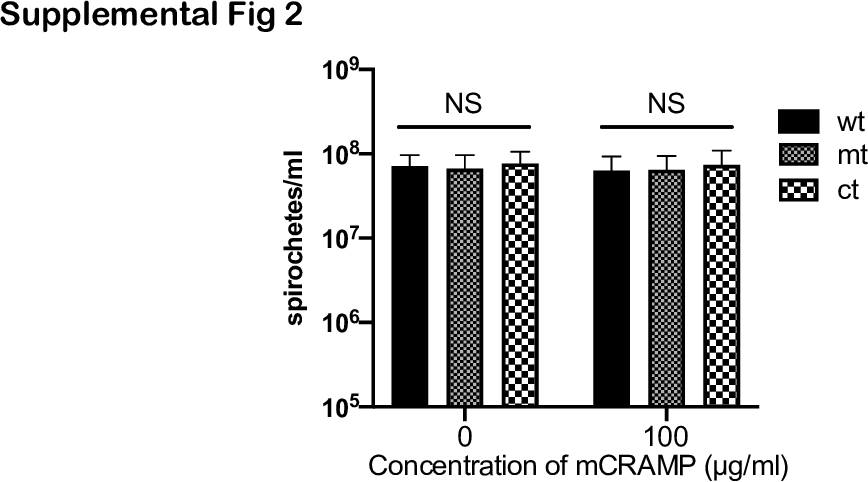

Supplement: S2 Fig — All three strains (wt, mt and ct) were propagated at 105/ml in BSKII growth medium at pH 6.8/32°C with 100 μg/ml of mCRAMP. Cells were enumerated every 24 hours using dark field microscopy. The cultures were grown in triplicate and one out of two independent experiments is shown. Statistical analysis of the difference in the number of wt and mt or ct and mt spirochetes was done by unpaired t test. (TIF) [file ppat.1009535.s002.tif]

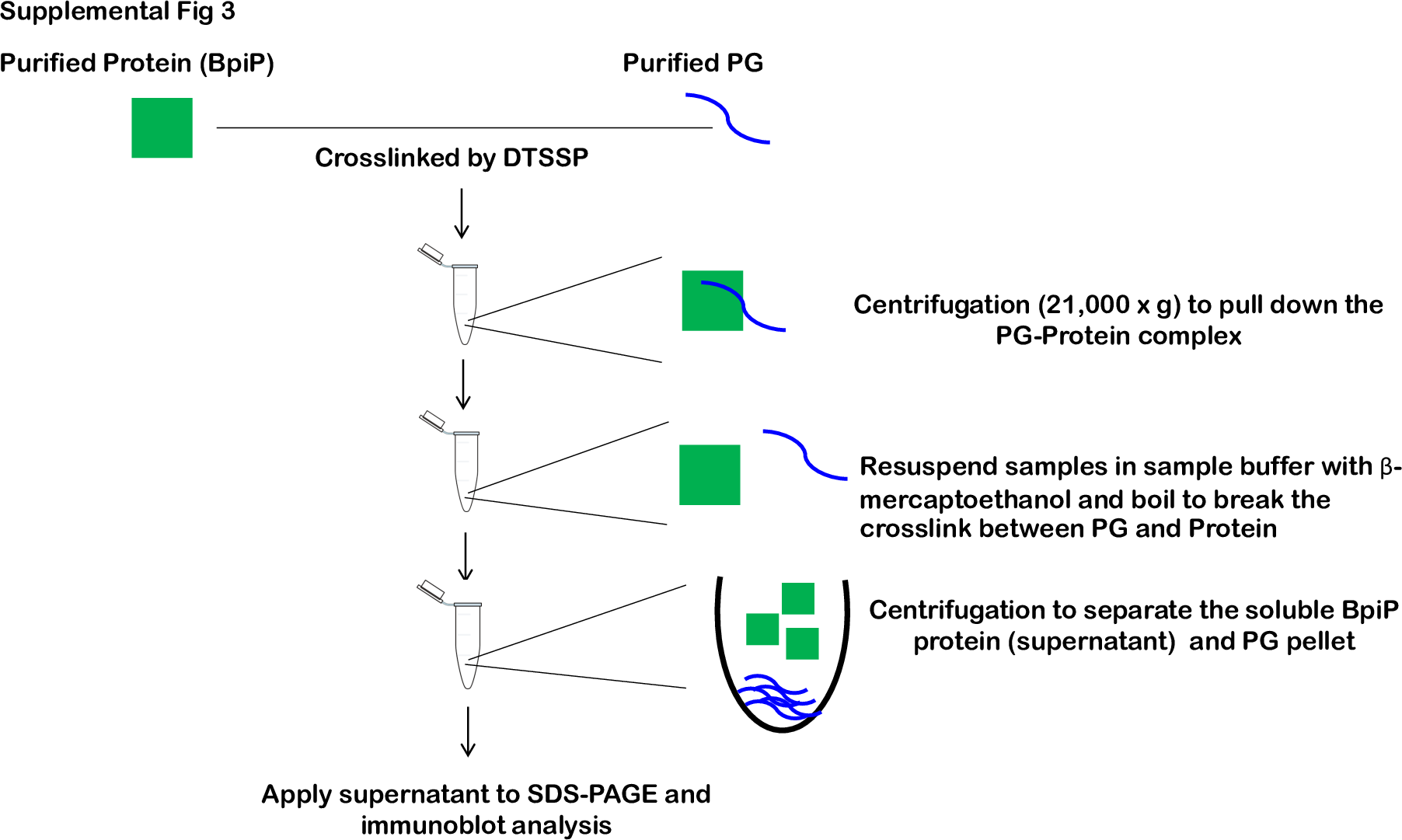

Supplement: S3 Fig — BpiP (wild type and site-specifically altered proteins) were crosslinked to purified PG (devoid of any bound proteins) using the crosslinker DTSSP. The complexes were pulled down by high speed centrifugation and the samples boiled to release the bound proteins and supernatant was separated from PG and analyzed for levels of BpiP by immunoblot analysis using anti-BpiP serum generated against C-terminal region of BpiP. (TIF) [file ppat.1009535.s003.tif]

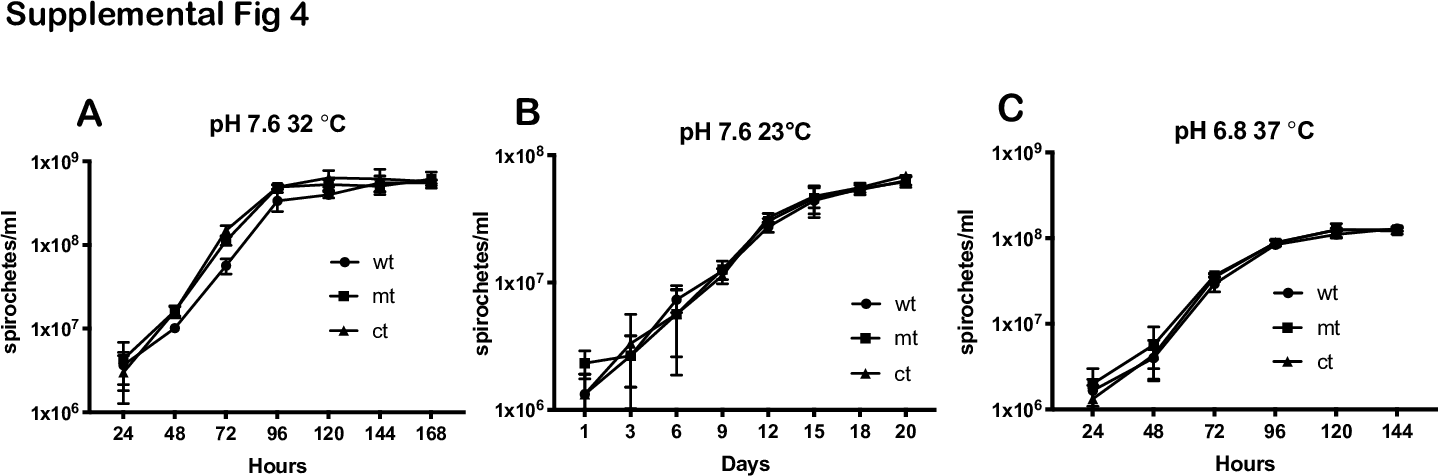

Supplement: S4 Fig — Wild type (B31/A3), mt and ct strains were diluted from stationary phase (1×10^8^ bacteria ml−1) cultures, re-seeded at 5×10^5^ bacteria ml−1 in BSKII medium and enumerated every 24 hours using dark field microscopy under different growth conditions. The cultures were grown in triplicate, with three independent trials. Error bars indicate standard error. Levels of significance were determined using two-way ANOVA with α = 95%. (TIF) [file ppat.1009535.s004.tif]
